# Supplementary material for: Psoriatic skin inflammation is promoted by c‐Jun/AP‐1‐dependent CCL2 and IL‐23 expression in dendritic cells
Source: EMBO Mol Med. 2021 Mar 16;13(4):e12409. doi: 10.15252/emmm.202012409 (PMC8033525; doi:10.15252/emmm.202012409)
Supplement: Supplementary file 1 — Appendix [file EMMM-13-e12409-s006.pdf]

Appendix of the research article entitled:

Psoriatic skin inflammation is promoted by c-Jun/AP-1 dependent CCL2 and IL-23 expression in Dendritic cells

Novoszel et al.

Table of contents:

Appendix Figure S1. Kinetic of keratinocyte differentiation and proliferation in IMQ-treated c-Jun$^{\Delta/\Delta}$CD11c-Cre mice

Appendix Figure S2. CCL2 is dispensable for IMQ-induced skin inflammation

Appendix Figure S3. AP-1 inhibition reduces CCL2 and IL-23 expression in IMQ-stimulated BMDCs

Appendix Figure S4. IL-23 is dispensable for pDC stimulation and migration to skin

Appendix Figure S5. c-Jun/AP-1 controls CCL-2 and IL-23 expression in BMDCs stimulated with the Adenine analogs and TLR7 ligands CL264 and CL307.

Appendix Figure S6. Expression analysis of cutaneous immune cells in steady-state.

Appendix Figure S7. Gating strategy for cutaneous immune cell populations

Appendix Figures

Appendix Methods

Appendix Table S1. Antibodies

Appendix Table S2. Primers

Appendix Table S3. List of *P* -values

Appendix Figure Legends

Appendix Figure S1. Kinetic of keratinocyte differentiation and proliferation in IMQ-treated c-Jun$^{\Delta/\Delta}$CD11c-Cre mice

A. Immunofluorescence of K5 (red), K10 (green) and DAPI in back skin from *c-Jun*$^{fl/fl}$ and *c-Jun*$^{\Delta/\Delta}$CD11c-Cre mice treated daily with IMQ for 36, 48 and 72h. Scale bar: 100 µm, Magnification 20x.

B. Immunofluorescence of Ki67 (green) and DAPI in back skin from *c-Jun*$^{fl/fl}$ and *c-Jun*$^{\Delta/\Delta}$*CD11c*-Cre mice treated daily with IMQ for 36, 48 and 72h. Scale bar: 100 µm, Magnification 20x.

Appendix Figure S2. CCL2 is dispensable for IMQ-induced skin inflammation

A. Hematoxylin and eosin stained sections of mouse back skin from wild-type and *Ccl2*$^{-/-}$ mice treated with IMQ for 3 and 5 days. Bright-field images, Magnification: 20x, Scale bar: 100 µm.

B. Epidermal thickness of the back skin was analyzed at the indicated time points in *Ccl2*$^{-/-}$ mice. (*n* = 3-21; 2 independent experiments).

C. Trans-epidermal water loss (TEWL) was analyzed in the back skin of wild-type and *Ccl2*$^{-/-}$ mice at the indicated time-points. (*n* = 3-9; 2 independent experiments).

D. Flow cytometric analysis of the immune infiltrate in back skin of wild-type and *Ccl2*$^{-/-}$ mice. Numbers next to gates give the percentage of indicated immune cells among percentage of live, single, CD45^{+} cells. Plots shown are pre-gated on live, single, CD45^{+} cells.

E. Quantification of immune cell populations as defined in (D). Results are given as percentage of live, single cells. (*n* = 6; 2 independent experiments).

Data information: Data are shown as mean ± SEM. *P*-values were calculated by unpaired, two-tailed *t*-test (E) or one-way (B) or two-way ANOVA with Tukey multiple comparison test (C). Statistical significance: ns > 0.05, **P* < 0.05, ***P* < 0.01. See Appendix Table S3 for exact *P*-values.

Appendix Figure S3. AP-1 inhibition reduces CCL2 and IL-23 expression in IMQ-stimulated BMDCs

A. BMDCs were generated from *Tlr7*$^{-/-}$, *c-Jun*$^{fl/fl}$ and *c-Jun*$^{\Delta/\Delta}$ *Mx1*-Cre BM, pre-treated with the AP-1 inhibitor T-5224 (20 µM, 1h) and/or stimulated with IMQ for 16h. Mean fluorescence intensity (MFI) of BMDC-CD80 was analyzed by flow cytometry.

(*n* = 2-3, 2 independent experiments performed (*n* = 2-6), one representative shown).

B. BMDCs were generated and analyzed for CD86 expression as described in (A).

C. BMDCs generated from *Tlr7*$^{-/-}$, *c-Jun*$^{fl/fl}$ or *c-Jun*$^{\Delta/\Delta}$ *Mx1*-Cre BM, were pre-treated with the AP-1 inhibitor T-5224 (20 µM, 1h) and/or stimulated with IMQ for 16h. CCL2 protein was quantified in supernatants by ELISA. (*n*= 2-6; 2 independent experiments).

D. BMDCs were generated as described in (C). 1x10^5 BMDCs were plated, pre-treated with the AP-1 inhibitor T-5224 (20 µM, 1h) and analyzed for IL-23 expression 4 h after stimulation with IMQ by ELISA.

Data information: Data are shown as mean ± SEM. *P*-values were calculated by one-way ANOVA with Tukey multiple comparison test (A-D). Statistical significance: ns > 0.05, **P* < 0.05, ***P* < 0.01, *** *P* <0.001, **** *P* <0.0001. See Appendix Table S3 for exact *P*-values.

Appendix Figure S4. IL-23 is dispensable for pDC stimulation and migration to skin

A. Flow cytometry of back skin 12h after intradermal (i.d.) injection of rIL-23 (1 µg). Analyzed were pDCs (BST-2$^+$B220$^+$CD11c^{int}CD11b$^-$). (*n* = 6; 2 independent experiment).

B. qRT-PCR detection of *Ccl2* and *Il17a* mRNA expression levels in total back skin of wild-type mice after i.d. injection of rIL-23 (1 µg; 6, 12 and 36h). (*n* = 4-11; 2 independent experiments).

C. qRT-PCR detection of *Il-12rb1*, *Il-12rb2* and *Il-23r* mRNA expression levels in FACS sort purified (B220$^+$CD11c$^+$CD11b$^-$; purity > 90%) BM-pDCs stimulated with IMQ for 8h. (*n* = 3; 3 independent experiments).

D. BM-pDCs were stimulated with rIL-12 (100 ng) or rIL-23 (100 ng) and/or IMQ for 8h. qRT-PCR detection was performed for *Gzmb*, *Trail* and *Ifng* mRNA. (*n*= 4-9; 3-4 independent experiments).

Data information: Data are shown as mean ± SEM. *P*-values were calculated by unpaired, two-tailed *t*-test (A, C and D) or one-way ANOVA with Dunnett´s multiple comparison test (B). ns > 0.05, **P* < 0.05, ***P* < 0.01, **** *P* <0.0001. See Appendix Table S3 for exact *P*-values.

Appendix Figure S5. c-Jun/AP-1 controls CCL-2 and IL-23 expression in BMDCs stimulated with the Adenine analogs and TLR7 ligands CL264 and CL307.

A. BMDCs were generated from *Tlr7*$^{-/-}$, *c-Jun*$^{fl/fl}$ and *c-Jun*$^{\Delta/\Delta}$ *Mx1*-Cre BM and stimulated with CL264 or CL307 for 16h. Mean fluorescence intensity (MFI) of CD80 was analyzed on BMDCs by flow cytometry. (*n* = 2-3, 2 independent experiments performed (*n* = 4-6), one representative shown).
B. BMDCs were generated and analyzed for CD86 expression as described in (A). (*n* = 3-4, 2 independent experiments performed (*n* = 4-6), one representative shown).
C. BMDCs were generated from BM of indicated genotype and stimulated with CL264 or CL307 for 16h. CCL2 protein was quantified in supernatant by ELISA. (*n*= 4-6; 2 independent experiments).
D. BMDCs were generated as described in (C). $1x10^5$ BMDCs were plated and analyzed for IL-23 expression 4 h after stimulation with CL264 or CL307 by ELISA.

Data information: Data are shown as mean ± SEM. *P*-values were calculated by one-way ANOVA with Tukey multiple comparison posttest (A-D). ns > 0.05, **P* < 0.05, ***P* < 0.01, *** *P* <0.001, **** *P* <0.0001. See Appendix Table S3 for exact *P*-values.

Appendix Figure S6. Expression analysis of cutaneous immune cells in steady-state.

A. qRT-PCR detection of *c-Jun*, *Ccl2* and *Il23p19* in indicated cell populations sorted from untreated back skin as described in Figure 4B. Fold change is shown relative to cDC1. (10 mice were pooled, 1 experiment).

B. qRT-PCR detection of *Il23p19* in granulocytes (Gr-1$^+$), non-immune cells (CD45$^-$) and T cells (CD3ε$^+$) sorted from untreated back skin as described in EV Figure 2E. (*n* = 4-5; 2 independent experiments).

Data information: Data are shown as mean ± SEM. *P*-values were calculated by one-way ANOVA with Tukey multiple comparison posttest (B). ns > 0.05, **P* < 0.05, ***P* < 0.01. See Appendix Table S3 for exact *P*-values.

Appendix Figure S7. Gating strategy for cutaneous immune cell populations

A. Gating strategy for flow cytometric analysis of immune cells in mouse back skin. In all experiments cell debris was discarded (FCS/SSC) and single events (SSC-A/SSC-W) were selected. Live cells (7-AAD) were gated on immune cells (CD45$^+$) before further analysis. Shown is a staining panel to identify dendritic cells (CD45$^+$CD11c$^+$MHCII$^+$CD64$^-$Ly6-C/G$^-$) negative (DC1) or positive (DC2) for CD11b, Macrophages (CD45$^+$CD11b$^+$CD64$^+$) negative (Mac1) or positive for MHCII (Mac2), Monocytes (CD45$^+$CD11b$^+$CD64$^-$Ly-6C^{hi}), neutrophils (CD45$^+$CD11b$^+$CD64$^-$Ly-6G$^+$), γδ T cells (CD45$^+$, CD11b$^-$, γδ TCR^{int}, CD3ε^{int}) and dendritic epidermal T cells (CD45$^+$, CD11b$^-$, γδ TCR$^+$,CD3ε$^+$). Ly6C$^+$ cells encompass CD45$^+$CD11b$^-$Ly6C$^+$ and CD11b$^+$ cells are CD11b$^+$, but negative for CD64, Ly6C and Ly6G. Color indicates overlap of cell population with populations defined by t-SNE in B.

B. t-SNE analysis was performed on a flow cytometry staining panel as described in A. Color in t-SNE plot on the left shows overlap with populations gated by conventional flow cytometry above. On the right a legend gives a label to the populations defined by t-SNE.

C. Protein quantification of CCL2, CXCL1, TNF-α, TSLP, IL-17A, IL-18 and IL-33 by Luminex multiplex assay in cutaneous lysates of vehicle or JNK inhibitor treated *c-Jun*$^{fl/fl}$ mice after 5 days of IMQ treatment on the back skin. (*n*= 3 for untreated group, 1 independent experiment and *n* = 6 for IMQ treated group, 2 independent experiments).

Data information: Data are shown as mean ± SEM. *P*-values were calculated by one-way ANOVA with Tukey multiple comparison test (C). ns > 0.05, $*P < 0.05$, $**P < 0.01$, $****P < 0.0001$ .See Appendix Table S3 for exact *P*-values.

Appendix Figure S1

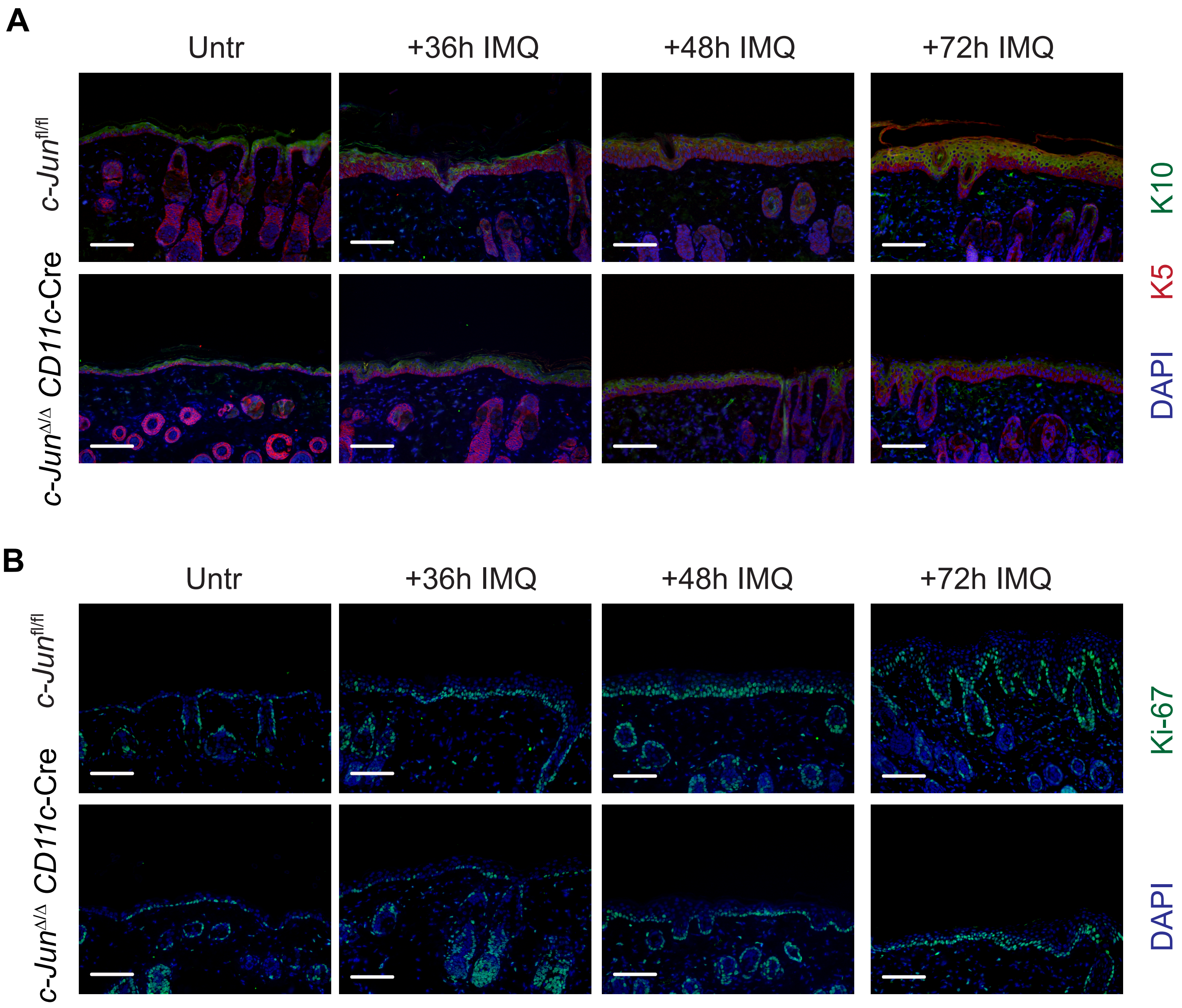

Appendix Figure S2

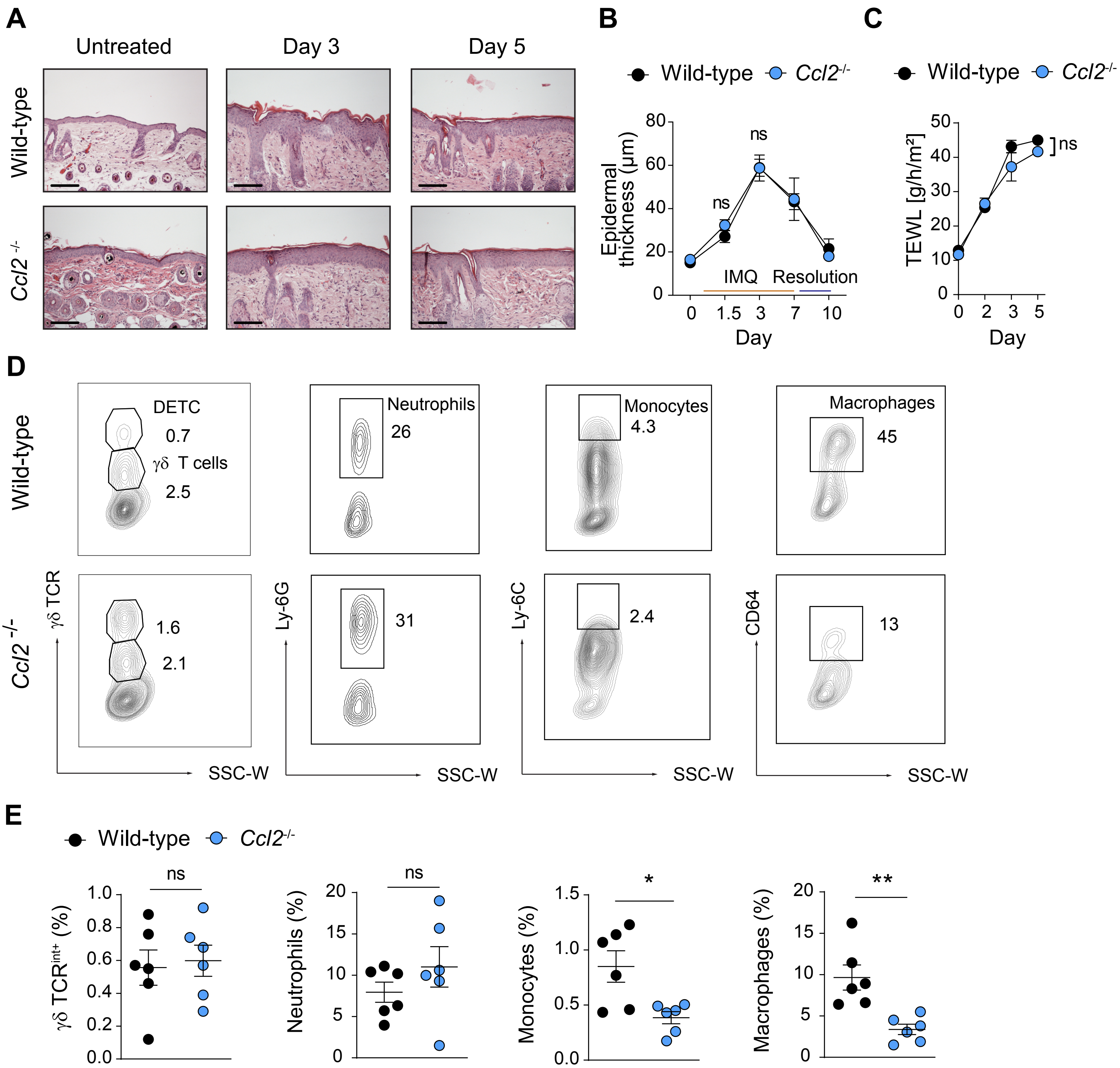

Appendix Figure S3

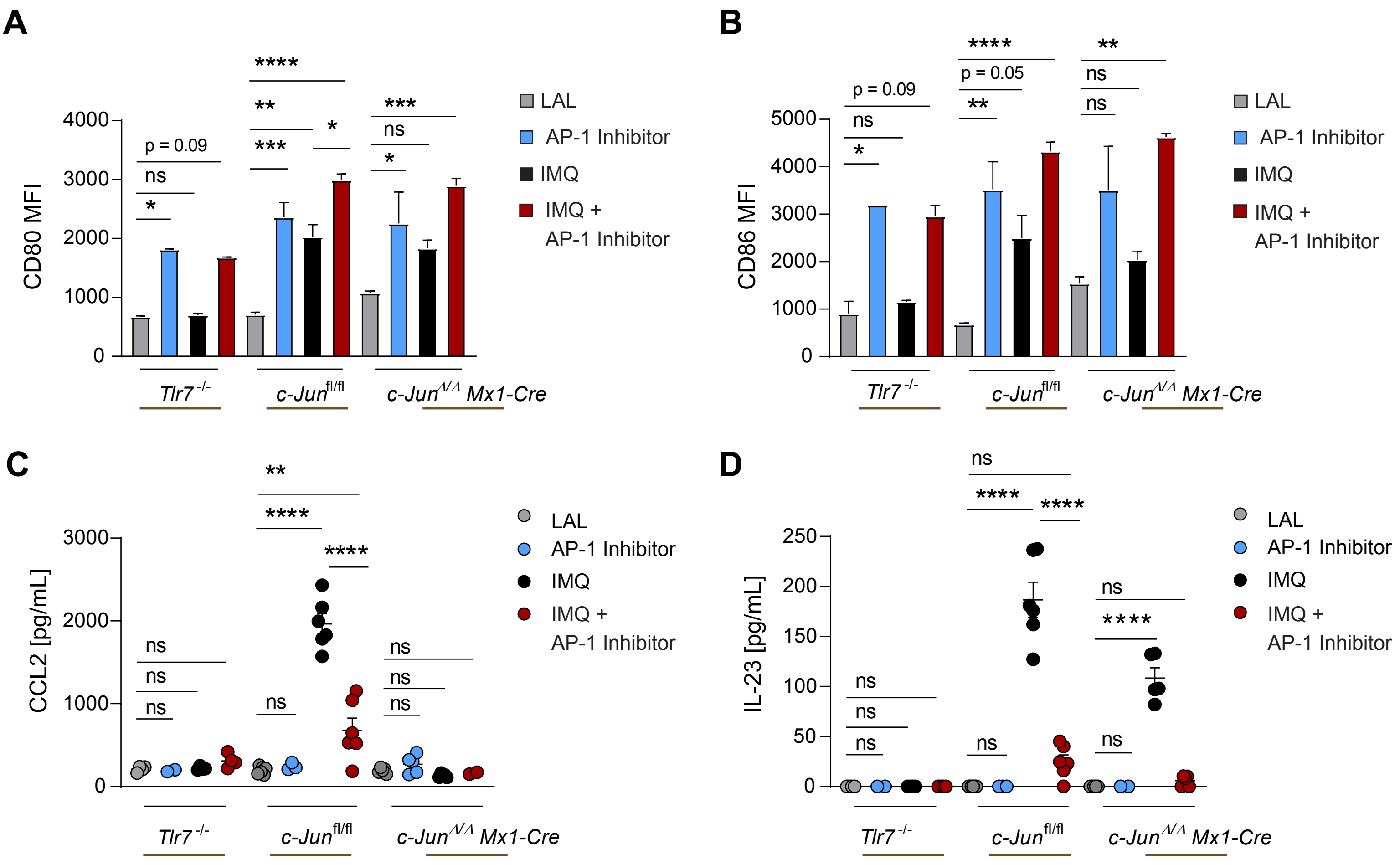

Appendix Figure S4

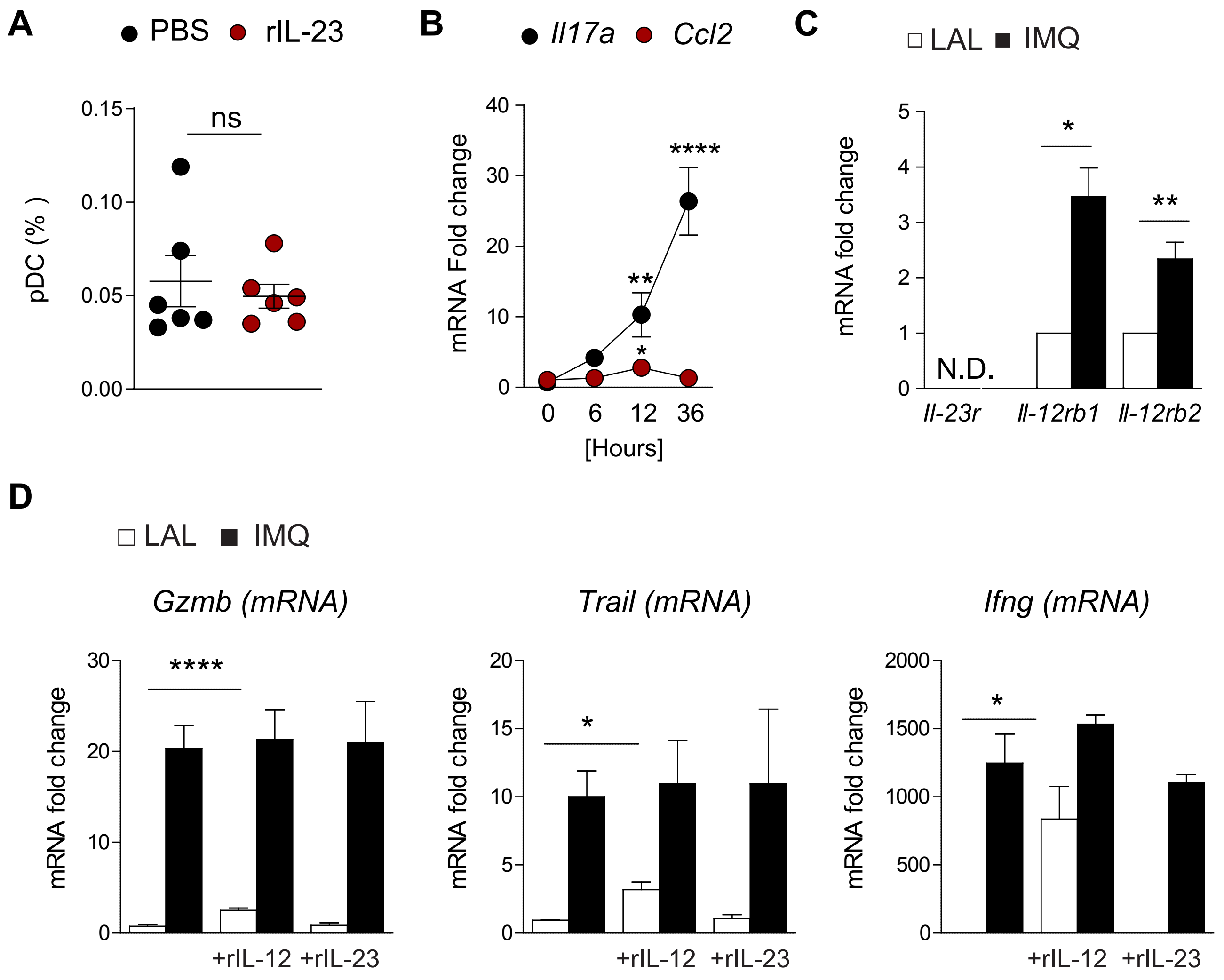

Appendix Figure S5

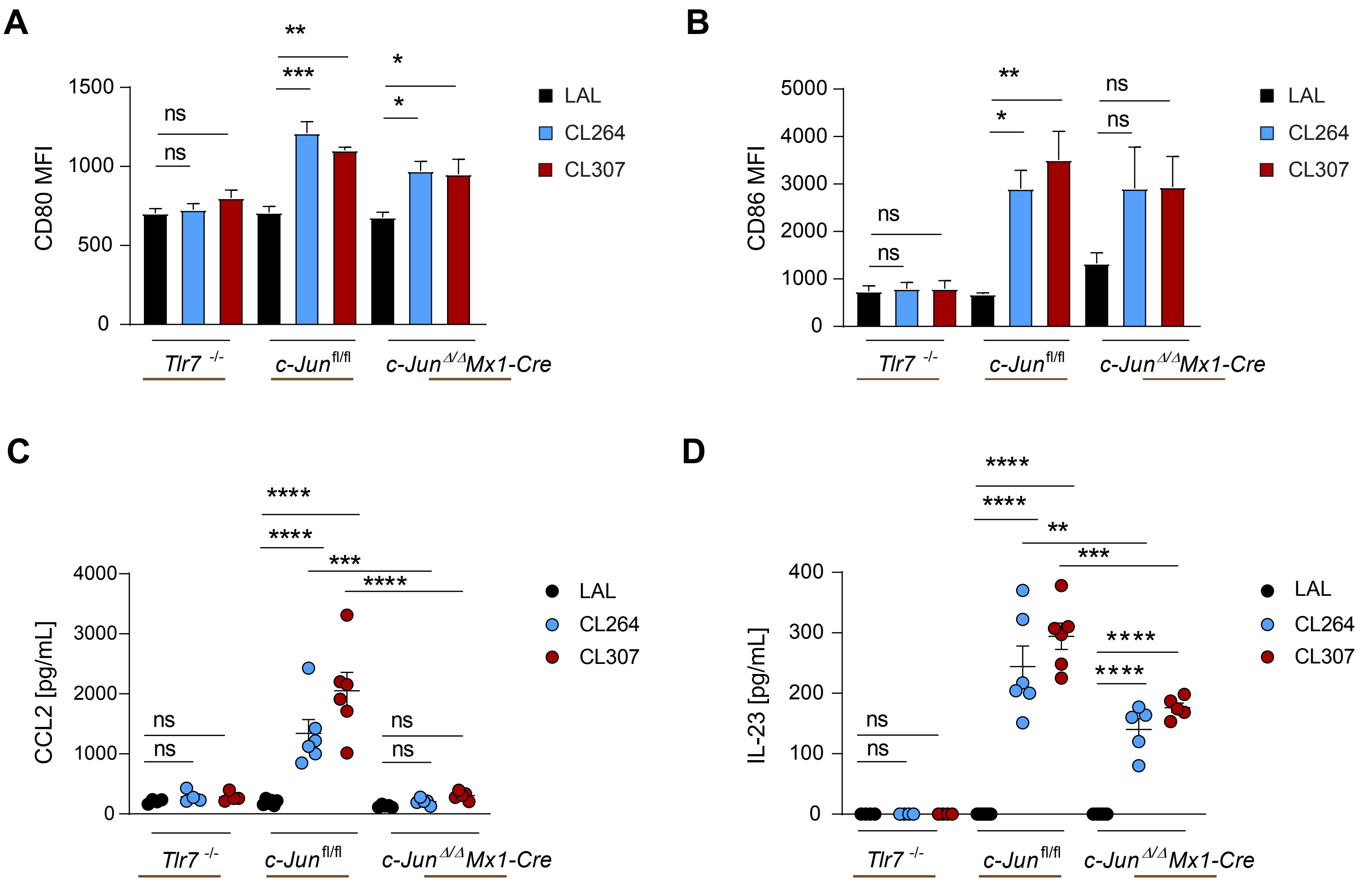

Appendix Figure S6

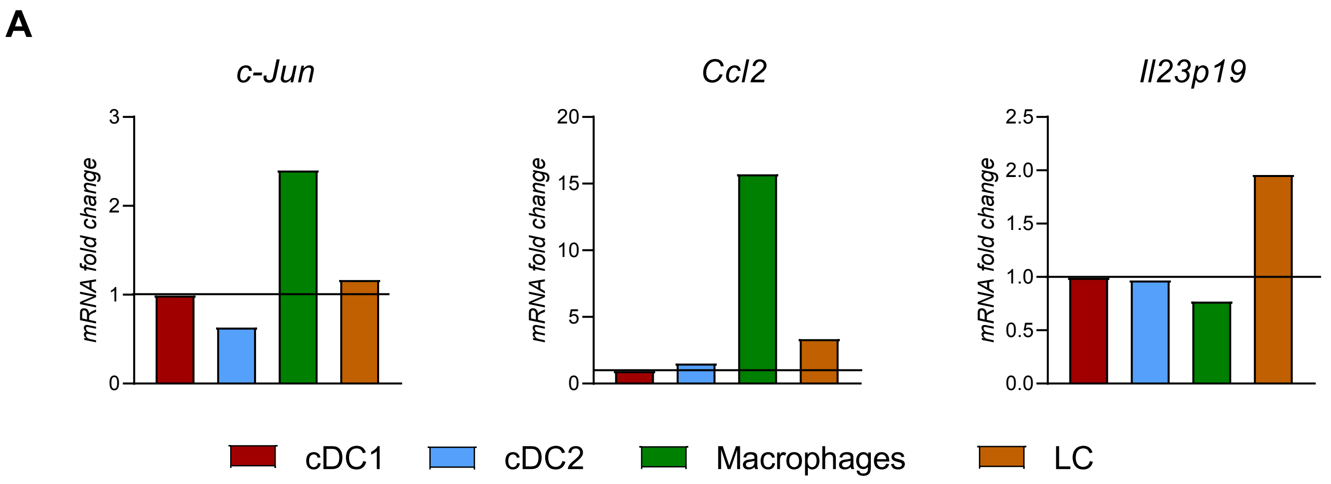

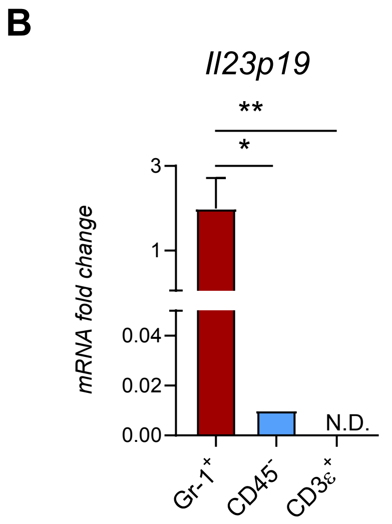

Appendix Figure S7

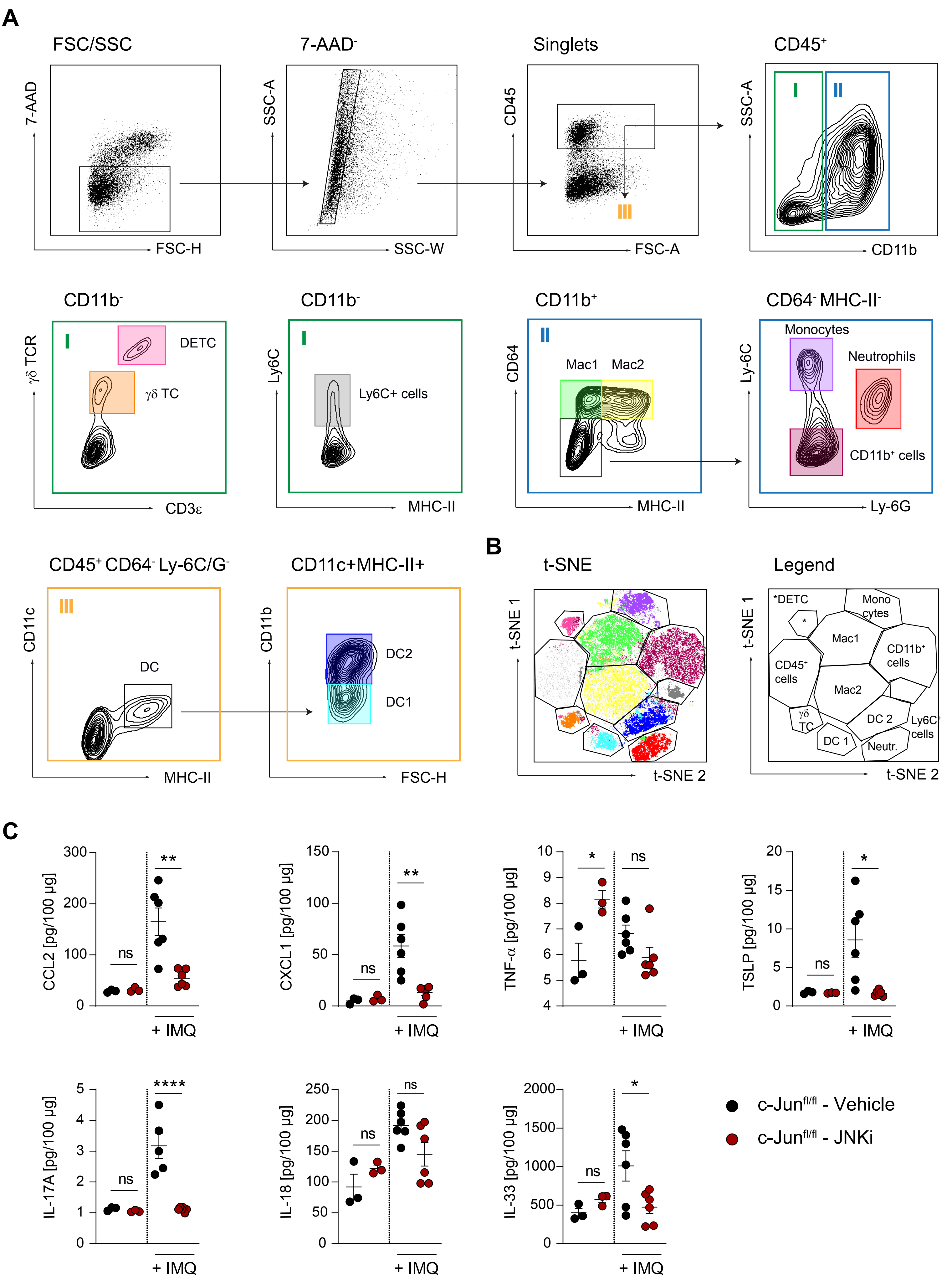

Appendix Methods

Preparation of cutaneous cell suspensions

To prepare a single cell suspension back skin was minced and digested in a mix containing PBS, 3% FCS (PAA), 100 µg/mL DNAse I (Sigma-Aldrich) and 100 µg/mL Liberase^{TM} (Roche) for 1h at 37°C. Enzymatically digested skin was washed and filtered through 70 µm cell strainers (BD Biosciences) before analysis.

For intracellular staining of IL-17A a single cell suspension was prepared from ears. Here, ears were separated in a dorsal and ventral side, minced and digested as described above.

Histological analysis

Skin tissue biopsies were fixed in formalin and embedded in paraffin. 5 µm sections were cut before sections were deparaffinized in xylene and rehydrated by ethanol (2 x 100 and 2 x 70 %). Sections were stained with hematoxylin and eosin (H&E) according to standard procedures. Epidermal thickness was determined as the length between basement membrane and stratum corneum. For each H&E stained section 3 pictures (20x, random position) were taken and for each picture epidermal thickness (4x measurement points) and epidermal layers was quantified using ImageJ.

Immunofluorescence of mouse tissue

For immunofluorescence of formalin-fixed murine back skin antigen retrieval (Citrate buffer pH =6, DAKO) was performed on deparaffinized skin sections. Sections were blocked with 5 % horse serum, 2 % bovine serum albumin (BSA) in phosphate-buffered saline (PBS) (blocking buffer) for 1 hour (room temperature). An overnight incubation at 4 °C with the primary antibodies (AB) diluted in blocking buffer followed. A washing step (3 x PBS, 5 min) and labelling with the appropriate secondary antibodies was done next (1h, dark humidified chamber). A counterstain with DAPI was performed. For immunofluorescence of frozen tissue, back skin was embedded in OCT (Sakura), followed by an acetone fixation (20 min, 4 °C). Lastly, for immunofluorescence of cell culture suspensions fixation in 4 % paraformaldehyde for 20 minutes was done. After fixation cells were smeared on poly-L-Lysine coated glass slides. Blocking and staining with primary and secondary AB followed as described above.

Primary AB used are listed in Appendix Table S1 and their fixation method is indicated. Images were recorded on a Nikon Eclipse 80i microscope.

Western blot

Western blot was done as previously described (Lichtenberger et al, 2010) with antibodies listed in Appendix Table S1. Western blots were automatically recorded with a ChemiDoc Touch Imaging System (Biorad).

Appendix Table S1. Antibodies

Reagent	Source	Identifier	Clone	Dilution
Flow cytometry antibodies				
B220	BioLegend	APC, PE-Cy7, BV650	RA3-6B2	¼ µg/10^6 cells
BST-2	BioLegend	PE	129C1	¼ µg/10^6 cells
BST-2	ThermoFisher	PE	eBio129c	¼ µg/10^6 cells
CD-3ε	BioLegend	Pe-Cy7	145-2C11	¼ µg/10^6 cells
CD11b	BioLegend	AF488, Pb, PE-Cy7	M1/70	¼ µg/10^6 cells
CD11c	BioLegend	AF488, APC, BV605	N418	¼ µg/10^6 cells
CD19	BioLegend	AF700	6D5	¼ µg/10^6 cells
CD45	BioLegend	PE, APC, APC-Cy7, AF700, BV 510	30-F11	¼ µg/10^6 cells
CD64	BioLegend	PE	X54-5/7.1	¼ µg/10^6 cells
CD80	BioLegend	PE	16-10A1	¼ µg/10^6 cells
CD86	BioLegend	APC-Cy7	GL-1	¼ µg/10^6 cells
CD103	BD Pharmingen	PE	M290	¼ µg/10^6 cells
CD172a	BioLegend	PE-Cy7	P84	¼ µg/10^6 cells
CD207	BioLegend	APC	4C7	¼ µg/10^6 cells
Gr-1	BioLegend	Pb	RB6-8C5	¼ µg/10^6 cells
Ly6C	BD Pharmingen	FITC	AL-21	¼ µg/10^6 cells
Ly6C	BioLegend	Pb, PE-Cy7, BV510	HK1.4	¼ µg/10^6 cells
Ly6G	BioLegend	Pb, APC-Cy7, AF700	1A8	¼ µg/10^6 cells
MHC-II	BioLegend	PE-Cy7, APC-Cy7	M5/114.15.2	¼ µg/10^6 cells
NK1.1	BioLegend	AF700	PK136	¼ µg/10^6 cells
TCRβ	BioLegend	AF700	H57-597	¼ µg/10^6 cells
XCR1	BioLegend	APC	ZET	¼ µg/10^6 cells
γδ TCR	BioLegend	FITC, APC	GL3	¼ µg/10^6 cells
Human FC antibodies				
CD1a	BD Biosciences	Pb	HI149	¼ µg/10^6 cells
CD80	BD Biosciences	Qdot 605	L307.4	¼ µg/10^6 cells
CD86	BD Biosciences	Pe-Cy7	2331	¼ µg/10^6 cells
Intracellular FC antibodies				
IL-17A	BioLegend	PE	TC11-18H10.1	¼ µg/10^6 cells
PE Rat IgG1, κ Isotype Ctrl	BioLegend	PE	RTK 207-1	¼ µg/10^6 cells
IL-23p19	ThermoFisher	efluor660	fc23cpg	¼ µg/10^6 cells
Rat IgG1, κ Isotype Ctrl, eFluor 660	ThermoFisher	efluor660	eBRG1	¼ µg/10^6 cells
IL-12p40	BioLegend	PE	C15.6	¼ µg/10^6 cells

Immunofluorescence (IF on Paraffin (P) or frozen (F) tissue and cell culture suspensions (S))				
K5 (IF-P)	Progen	GP-CK5	guinea pig polyclonal	1:100
K10 (IF-P)	BioLegend	905401	Poly19054	1:100
Ki-67 (IF-P)	Abcam	ab15580	Rabbit polyclonal	1:100
c-Jun (IF-P)	Cell Signaling	9165	60A8	1:100
CD11c (IF-F | IF-S)	BioLegend	117311	N418	1:400
IL23p19 (IF-S)	Abcam	45420	Rabbit polyclonal	1:100
CD11c (Human) (IF-F)	BD Biosciences	550375	B-ly6	1:50
CCL2 (Human) (IF-F)	Sigma-Aldrich	HPA019163-100UL	HPA019163	1:100
IL23p19 (Human) (IF-F)	Sigma Aldrich	HPA001554- 100UL	HPA001554	1:100
c-Jun (Human) (IF-F)	Biotechne	AF2670	Sheep polyclonal	1:50
CD1a (Human) (IF-P)	Novus biologicals	NBP2-34313	O10	1:50
CD1c (Human) (IF-P | IF-F)	Novus biologicals	NB100-65313	L161	1:100
CD14 (Human) ((IF-P | IF-F)	BioLegend	325603	HCD14	1:100

Chromatin Immunoprecipitation				
c-Jun	Cell Signaling	9165	60A8	1:50

Western Blot				
c-Jun	Cell Signaling	9165	60A8	1:1000
P-c-Jun	Cell Signaling	3270	D47G9	1:1000
JNK/SAPK	Cell Signaling	9252	-	1:1000
P-JNK/P-SAPK	Cell Signaling	9255	G9	1:1000
Vinculin	Sigma Aldrich	V9131	hVIN-1	1:500

Appendix Table S2. Primers

Primer name	Forward primer (5´-3´)	Reverse primer (5´-3´)
c-Jun	AAAACCTTGAAAGCGCAAAA	CGCAACCAGTCAAGTTCTCA
c-Fos	CAGCCTTTCCTACTACCATTCC	ACAGATCTGCGCAAAAGTCC
Ccl2	GAAGGAATGGGTCCAGACAT	ACGGGTCAACTTCACATTCA
Ccl20	CGACTGTTGCCTCTCGTACA	GAGGAGGTTCACAGCCCTTT
Cxcl1	GCCTATCGCCAATGAGCTG	ATTCTTGAGTGTGGCTATGA
Fosb	GTTCGCAGAGAGCGGAAC	GCCTTTTCCTCTTCAAGCTG
Fra-1	GCATGTACCGAGACTACGGG	AGGTGGAACTTCTGCTGCTG
Fra-2	ACGCCGAGTCCTACTCCAG	CAGGCATATCTACCCGGAAC
Gzmb	ATTCCCCACCCAGACTATAATCC	TTACTCTTCAGCTTTAGCAGCATGA
Il1b	GGGCCTCAAAGGAAAGAATC	TACCAGTTGGGGAACTCTGC
Il6	GAGGATACCACTCCCAACAGACC	AAGTGCATCATCGTTGTTCATACA
Il12rb1	ACTGGAATGTGTCTGAAG	CGTATCTGGATCTCTTGG
Il12rb2	CCTCAATGGTATAGCAGAAC	TAGCCTTGGAATCCTTGG
Il17a	GCTCCAGAAGGCCCTCAGA	CTTTCCCTCCGCATTGACA
Il22	TTTCCTGACCAAACTCAGCA	CTGGATGTTCTGGTCGTCAC
Il23p19	ACCAGCGGGACATATGAATCTA	GAACTGGCTGTTGTCCTTGA
Il23r	GCTCGGATTTGGTATAAAGG	ACTTGGTATCTATGTAGGTAGG
Ifng	CTGAGACAATGAACGCTACAC	TCCACATCTATGCCACTTGAG
Junb	ATGTGCACGAAAATGGAACA	CCTGACCCGAAAAGTAGCTG
S100a8	TTCGTGACAATGCCGTCTGA	AGGGCATGGTGATTTCCTTGT
S100a9	AGATGGCCAACAAAGCACCT	TCTCTTTCTTCATAAAGGTTGCCA
Tbp	GGGGAGCTGTGATGTGAAGT	CCAGGAAATAATTCTGGCTCAT
Tnfa	CTGAACTTCGGGGTGATCGG	GGCTTGTCACTCGAATTTTGAGA
Trail	GTGTCTGTGGCTGTGACTTACA	AATGCCCTTTCCGAGAGGA
**Promoter**		
c-Jun (CHIP)	CTCTAGCCACAACAACCTCA	TTCACACTAGCAGGTGACTC
mut1(Luciferase Assay)	TCCCACCTGCTCTGAGCCA CCTGCTAGTGTGAAG	TTCCCCTCCCTACATCA TCTCCCTCTTTGGAGAG
mut2 (Luciferase assay)	GGGGAATCCCACCTGCTC ATAGTCA CCTGCTAGTGTGA	TCCCTACATCATCTCCCTCTTT GGAGAGCCCAGGTATG

Appendix Table S3. List of *P* -values

Figure 1

**Figure 1 A**		
Tukey's multiple comparisons test	Summary	Adjusted P Value
c-Jun $^{fl/fl}$- 1.5 days **vs** c-Jun $^{\Delta/\Delta}$ *Mx1*-Cre - 1.5 days	*	0.0218
c-Jun $^{fl/fl}$- 3 days **vs** c-Jun $^{\Delta/\Delta}$ *Mx1*-Cre - 3 days	***	0.0007
**Figure 1B**		
Tukey's multiple comparisons test	Summary	Adjusted P Value
c-Jun $^{fl/fl}$- 1.5 days **vs** c-Jun $^{\Delta/\Delta}$ *CD11c*-Cre - 1.5 days	***	0.0006
c-Jun $^{fl/fl}$- 3 days **vs** c-Jun $^{\Delta/\Delta}$ *CD11c*-Cre - 3 days	****	<0,0001
**Figure 1C**		
Tukey's multiple comparisons test	Summary	Adjusted P Value
c-Jun $^{fl/fl}$- 1.5 days **vs** c-Jun $^{\Delta/\Delta}$ *K5*-Cre-ER^{T2} - 1.5 days	ns	>0,9999
c-Jun $^{fl/fl}$- 3 days **vs** c-Jun $^{\Delta/\Delta}$ *K5-Cre*-ER^{T2}-Cre - 3 days	ns	0.9972
**Figure 1 E LEFT**		
Tukey's multiple comparisons test	Summary	Adjusted P Value
c-Jun $^{fl/fl}$- 2 days **vs** c-Jun $^{\Delta/\Delta}$ *CD11c*-Cre - 2 days	*	0.018
c-Jun $^{fl/fl}$- 3 days **vs** c-Jun $^{\Delta/\Delta}$ *CD11c*-Cre - 3 days	****	<0,0001
c-Jun $^{fl/fl}$- 5 days **vs** c-Jun $^{\Delta/\Delta}$ *CD11c*-Cre - 5 days	****	<0,0001
**Figure 1 E RIGHT**		
Tukey's multiple comparisons test	Summary	Adjusted P Value
c-Jun $^{fl/fl}$- 2 days **vs** c-Jun $^{\Delta/\Delta}$ *CD11c*-Cre - 2 days	****	<0,0001

c-Jun $^{fl/fl}$- 3 days **vs** c-Jun $^{\Delta/\Delta}$ *CD11c*-Cre - 3 days	****	<0,0001
c-Jun $^{fl/fl}$- 5 days **vs** c-Jun $^{\Delta/\Delta}$ *CD11c*-Cre - 5 days	****	<0,0001
	**Figure 1 F**	
Bonferroni's multiple comparisons test*	Summary	Adjusted P Value
c-Jun $^{fl/fl}$- 0 days **vs** c-Jun $^{\Delta/\Delta}$ *CD11c*-Cre - 0 days	ns	>0,9999
c-Jun $^{fl/fl}$- 0 days **vs** *c-Jun* $^{fl/fl}$- 2 days	ns	0.8893
c-Jun $^{fl/fl}$- 0 days **vs** *c-Jun* $^{fl/fl}$- 3 days	***	0.0002
c-Jun $^{fl/fl}$- 0 days **vs** *c-Jun* $^{fl/fl}$- 5 days	****	<0,0001
c-Jun $^{fl/fl}$- 2 days **vs** c-Jun $^{\Delta/\Delta}$ *CD11c*-Cre - 2 days	ns	>0,9999
c-Jun $^{fl/fl}$- 3 days **vs** c-Jun $^{\Delta/\Delta}$ *CD11c*-Cre - 3 days	**	0.0011
c-Jun $^{fl/fl}$- 5 days **vs** c-Jun $^{\Delta/\Delta}$ *CD11c*-Cre - 5 days	*	0.038
	**Figure 1 G**	
Bonferroni's multiple comparisons test*	Summary	Adjusted P Value
c-Jun $^{fl/fl}$- 0 days **vs** c-Jun $^{\Delta/\Delta}$ *CD11c*-Cre - 0 days	*ns*	*>0,9999*
c-Jun $^{fl/fl}$- 0 days **vs** *c-Jun* $^{fl/fl}$- 2 days	*	*0.0288*
c-Jun $^{fl/fl}$- 0 days **vs** *c-Jun* $^{fl/fl}$- 3 days	****	*<0,0001*
c-Jun $^{fl/fl}$- 0 days **vs** *c-Jun* $^{fl/fl}$- 5 days	****	*<0,0001*
c-Jun $^{fl/fl}$- 2 days **vs** c-Jun $^{\Delta/\Delta}$ *CD11c*-Cre - 2 days	*ns*	*0.7809*
c-Jun $^{fl/fl}$- 3 days **vs** c-Jun $^{\Delta/\Delta}$ *CD11c*-Cre - 3 days	****	*<0,0001*
c-Jun $^{fl/fl}$- 5 days **vs** c-Jun $^{\Delta/\Delta}$ *CD11c*-Cre - 5 days	*	*0.0107*

Figure 1 H		
Bonferroni's multiple comparisons test*	Summary	Adjusted P Value
c-Jun $^{fl/fl}$- 0 days **vs** c-Jun $^{Δ/Δ}$ *CD11c*-Cre - 0 days	ns	>0,9999
c-Jun $^{fl/fl}$- 0 days **vs** *c-Jun* $^{fl/fl}$- 2 days	**	0.0024
c-Jun $^{fl/fl}$- 0 days **vs** *c-Jun* $^{fl/fl}$- 3 days	****	<0,0001
c-Jun $^{fl/fl}$- 0 days **vs** *c-Jun* $^{fl/fl}$- 5 days	****	<0,0001
c-Jun $^{fl/fl}$- 2 days **vs** c-Jun $^{Δ/Δ}$ *CD11c*-Cre - 2 days	ns	0.8749
c-Jun $^{fl/fl}$- 3 days **vs** c-Jun $^{Δ/Δ}$ *CD11c*-Cre - 3 days	ns	0.0624
c-Jun $^{fl/fl}$- 5 days **vs** c-Jun $^{Δ/Δ}$ *CD11c*-Cre - 5 days	***	0.0006

Figure 2

Figure 2A		
Bonferroni's multiple comparisons test	Summary	Adjusted *P* Value
c-Jun $^{fl/fl}$ -0 days **vs** *c-Jun* $^{Δ/Δ}$ *CD11c*-Cre- 0 days	ns	>0,9999
c-Jun $^{fl/fl}$ -0 days **vs** *c-Jun fl/fl -0.5 days*	****	<0,0001
c-Jun $^{fl/fl}$ -0 days **vs** *c-Jun fl/fl -1.5 days*	****	<0,0001
c-Jun $^{fl/fl}$ -0 days **vs** *c-Jun fl/fl -3 days*	ns	>0,9999
c-Jun $^{fl/fl}$ -0 days **vs** *c-Jun fl/fl -5 days*	ns	>0,9999
c-Jun $^{fl/fl}$- 0.5 days **vs** *c-Jun* $^{Δ/Δ}$ *CD11c*-Cre - 0.5 days	****	<0,0001
c-Jun $^{fl/fl}$- 1.5 days **vs** *c-Jun* $^{Δ/Δ}$ *CD11c*-Cre - 1.5 days	****	<0,0001
c-Jun $^{fl/fl}$- 3 days **vs** *c-Jun* $^{Δ/Δ}$ *CD11c*-Cre - 3 days	ns	>0,9999
c-Jun $^{fl/fl}$- 5 days **vs** *c-Jun* $^{Δ/Δ}$ *CD11c*-Cre - 5 days	ns	>0,9999

**Figure 2C**		
Multiple t-Test- Holm Sidak Method	Summary	Adjusted *P* Value
c-Jun $^{fl/fl}$- 0 days **vs** c-Jun $^{\Delta/\Delta}$ *CD11c*-Cre - 0 days	ns	0.7671
c-Jun $^{fl/fl}$- 0.5 days **vs** *c-Jun* $^{\Delta/\Delta}$ *CD11c-Cre* - 0.5 days	*	0.0354
**Figure 2D**		
Tukey's multiple comparisons test	Summary	Adjusted *P* Value
c-Jun $^{fl/fl}$- 0.5 days **vs** *c-Jun* $^{\Delta/\Delta}$ *CD11c*-Cre - 0.5 days	****	<0,0001
c-Jun $^{fl/fl}$- 1.5 days **vs** *c-Jun* $^{\Delta/\Delta}$ *CD11c*-Cre - 1.5 days	*	0.0109
**Figure 2E**		
Kruskal-Wallis-Test*	Summary	Adjusted *P* Value
c-Jun $^{fl/fl}$- LAL **vs** *c-Jun* $^{fl/fl}$ - IMQ	**	0.0087
c-Jun $^{fl/fl}$- IMQ **vs** *c-Jun* $^{\Delta/\Delta}$ *CD11c*-Cre - IMQ	***	0.0001
**Figure 2F**		
Tukey's multiple comparisons test	Summary	Adjusted *P* Value
c-Jun $^{fl/fl}$- LAL **vs** *c-Jun* $^{fl/fl}$ - IMQ	*	0.0404
c-Jun $^{fl/fl}$- IMQ **vs** *c-Jun* $^{\Delta/\Delta}$ *CD11c*-Cre - IMQ	****	<0,0001
**Figure 2G**		
Tukey's multiple comparisons test	Summary	Adjusted *P* Value
c-Jun $^{fl/fl}$- IMQ **vs** *Tlr7* $^{-/-}$ - IMQ	****	<0,0001
c-Jun $^{fl/fl}$- IMQ **vs** c-*Jun* $^{fl/fl}$- *IMQ*- JNKi	****	<0,0001
c-Jun $^{fl/fl}$- IMQ **vs** *c-Jun* $^{\Delta/\Delta}$ *Mx1*-Cre - IMQ	****	<0,0001
**Figure 2J - I**		
Tukey's PBS **vs** rCCL2	Summary	Adjusted *P* Value
c-Jun $^{fl/fl}$- PBS **vs** *c-Jun* $^{fl/fl}$ - rCCL2	*	0.0454

c-Jun $^{fl/fl}$- rCCL2 **vs** *c-Jun* $^{\Delta/\Delta}$ *CD11c*-Cre - rCCL2	ns	0.9246
**Figure 2J - II**		
Tukey's PBS **vs** IMQ	Summary	Adjusted *P* Value
c-Jun $^{fl/fl}$- PBS **vs** *c-Jun* $^{fl/fl}$ - IMQ	****	<0,0001
c-Jun $^{fl/fl}$- IMQ **vs** *c-Jun* $^{\Delta/\Delta}$ *CD11c*-Cre - IMQ	**	0.0084
**Figure 2J - III**		
Tukey's IMQ **vs** IMQ + rCCL2	Summary	Adjusted *P* Value
c-Jun $^{fl/fl}$- PBS **vs** *c-Jun* $^{fl/fl}$ - IMQ + rCCL2	**	0.0033
c-Jun $^{fl/fl}$- IMQ **vs** *c-Jun* $^{\Delta/\Delta}$ *CD11c*-Cre - IMQ + rCCL2	ns	0.8904

Figure 3

**Figure 3A**		
Unpaired t test with Welch's correction	Summary	*P* Value
c-Jun $^{fl/fl}$- LAL **vs** *c-Jun* $^{fl/fl}$ - IMQ	*	0.0236
JunB $^{fl/fl}$- LAL **vs** *JunB* $^{fl/fl}$ - IMQ	ns	0.1744
Fra-1 $^{fl/fl}$- LAL **vs** *Fra-1* $^{fl/fl}$ - IMQ	**	0.0023
Fra-2 $^{fl/fl}$- LAL **vs** *Fra-2* $^{fl/fl}$ - IMQ	**	0.0033
FosB $^{fl/fl}$- LAL **vs** *FosB* $^{fl/fl}$ - IMQ	****	<0,0001
Unpaired t test	Summary	*P* Value
c-Fos $^{fl/fl}$- LAL **vs** *c-Fos* $^{fl/fl}$ - IMQ	***	0.001
**Figure 3B**		
Tukey's multiple comparisons test	Summary	Adjusted P Value
c-Jun $^{fl/fl}$- **vs** c-Jun $^{\Delta/\Delta}$ *CD11c*-Cre	***	0.0003
c-Jun $^{fl/fl}$- **vs** *c-Jun* $^{fl/fl}$ - IMQ	***	0.0005
c-Jun $^{fl/fl}$-IMQ **vs** *c-Jun* $^{\Delta/\Delta}$ *CD11c*-Cre-IMQ	****	<0,0001

Figure 3C-*Il23p19*		
Unpaired t test with Welch's correction	Summary	*P* Value
c-Jun $^{fl/fl}$-32h **vs** c-Jun $^{\Delta/\Delta}$ CD11c-Cre-32h	*	0.0195
c-Jun $^{fl/fl}$-48h **vs** c-Jun $^{\Delta/\Delta}$ CD11c-Cre-48h	*	0.0109
**Figure 3C-*Il17a***		
Unpaired t test with Welch's correction	Summary	*P* Value
c-Jun $^{fl/fl}$-32h **vs** c-Jun $^{\Delta/\Delta}$ CD11c-Cre-32h	**	0.0096
c-Jun $^{fl/fl}$-48h **vs** c-Jun $^{\Delta/\Delta}$ CD11c-Cre-48h	*	0.049
**Figure 3D - IL-23**		
Tukey's multiple comparisons test	Summary	Adjusted P Value
c-Jun $^{fl/fl}$ **vs** c-Jun $^{\Delta/\Delta}$ CD11c-Cre	ns	0.9875
c-Jun $^{fl/fl}$-48h **vs** c-Jun $^{\Delta/\Delta}$ CD11c-Cre-48h	*	0.046
**Figure 3D - IL-17A**		
Kruskal-Wallis multiple comparison test	Summary	Adjusted P Value
c-Jun $^{fl/fl}$ **vs** c-Jun $^{\Delta/\Delta}$ CD11c-Cre	ns	*>0,9999*
c-Jun $^{fl/fl}$-48h **vs** c-Jun $^{\Delta/\Delta}$ CD11c-Cre-48h	*	0.0414
**Figure 3E**		
Tukey's multiple comparisons test	Summary	Adjusted *P* Value
c-Jun $^{fl/fl}$- IMQ **vs** *Tlr7*$^{-/-}$ - IMQ	****	<0,0001
c-Jun $^{fl/fl}$- IMQ **vs** c-*Jun* $^{fl/fl}$- *IMQ- JNKi*	***	0.0008
c-Jun $^{fl/fl}$- IMQ **vs** *c-Jun* $^{\Delta/\Delta}$ *Mx1*-Cre - IMQ	*	0.0199
**Figure 3F LEFT**		
Tukey's multiple comparisons test	Summary	Adjusted *P* Value

c-Jun $^{fl/fl}$ **vs** *c-Jun* $^{fl/fl}$- *IMQ*	****	<0,0001
c-Jun $^{fl/fl}$- IMQ **vs** *c-Jun* $^{\Delta/\Delta}$ *Mx1*-Cre - IMQ	****	<0,0001
**Figure 3F RIGHT**		
Tukey's multiple comparisons test	Summary	Adjusted *P* Value
c-Jun $^{fl/fl}$ **vs** c-Jun $^{fl/fl}$- IMQ	****	<0,0001
c-Jun $^{fl/fl}$- IMQ **vs** c-Jun $^{\Delta/\Delta}$ Mx1-Cre - IMQ	***	0.0005
**Figure 3G**		
Tukey's multiple comparisons test	Summary	Adjusted *P* Value
c-Jun $^{fl/fl}$ **vs** *c-Jun* $^{fl/fl}$- *IMQ*	*	0.0267
c-Jun $^{fl/fl}$- IMQ **vs** *c-Jun* $^{\Delta/\Delta}$ *CD11c*-Cre - IMQ	**	0.0056
**Figure 3I**		
Tukey's multiple comparisons test	Summary	Adjusted *P* Value
c-Jun $^{fl/fl}$ **vs** *c-Jun* $^{\Delta/\Delta}$ *Mx1*-Cre	ns	0.9792
c-Jun $^{fl/fl}$ **vs** *c-Jun* $^{fl/fl}$- IMQ	***	0.0003
c-Jun $^{fl/fl}$- IMQ **vs** *c-Jun* $^{\Delta/\Delta}$ *Mx1*-Cre - IMQ	***	0.0001
**Figure 3J**		
Tukey's multiple comparisons test	Summary	Adjusted P Value
LAL vs IMQ	****	<0,0001
IMQ vs IMQ + JNKi	****	<0,0001
IMQ vs mut1-IMQ	****	<0,0001
IMQ vs mut2-IMQ	**	0.0042
**Figure 3K**		
Tukey's multiple comparisons test	Summary	Adjusted *P* Value
c-Jun $^{fl/fl}$ **vs** c-Jun $^{fl/fl}$- *IMQ*	***	0.0006
c-Jun $^{fl/fl}$- IMQ **vs** *c-Jun* $^{\Delta/\Delta}$ *CD11c*-Cre - IMQ	*	0.0143

Figure 3L		
Tukey's multiple comparisons test	Summary	Adjusted *P* Value
c-Jun $^{fl/fl}$ **vs** c-*Jun* $^{fl/fl}$- *IMQ*	****	<0,0001
c-Jun $^{fl/fl}$ **vs** c-*Jun* $^{fl/fl}$- *IMQ + rIL-23*	****	<0,0001
c-Jun $^{fl/fl}$ **vs** c-*Jun* $^{fl/fl}$- *rIL-23*	***	0.0002
c-Jun $^{fl/fl}$- IMQ **vs** *c-Jun* $^{\Delta/\Delta}$ *CD11c*-Cre - IMQ	****	<0,0001
c-Jun $^{fl/fl}$- IMQ-IL-23 **vs** *c-Jun* $^{\Delta/\Delta}$ *CD11c*-Cre - IMQ-IL-23	ns	0.9294
c-Jun $^{fl/fl}$- IL-23 **vs** *c-Jun* $^{\Delta/\Delta}$ *CD11c*-Cre - IL-23	ns	>0,9999

Figure 4

Figure 4C- cDC2 - c-Jun		
Unpaired t test with Welch's correction	Summary	*P* Value
c-Jun $^{fl/fl}$- IMQ **vs** *c-Jun* $^{\Delta/\Delta}$ *CD11c*-Cre - IMQ	*	0.018
**Figure 4C- cDC2 - Ccl2**		
Unpaired t test	Summary	*P* Value
c-Jun $^{fl/fl}$- IMQ **vs** *c-Jun* $^{\Delta/\Delta}$ *CD11c*-Cre - IMQ	*	0.0348
**Figure 4C- cDC2 - Il23p19**		
Unpaired t test with Welch's correction	Summary	*P* Value
c-Jun $^{fl/fl}$- IMQ **vs** *c-Jun* $^{\Delta/\Delta}$ *CD11c*-Cre - IMQ	*	0.0359
**Figure 4C- Mac - *c-Jun***		
Unpaired t test	Summary	*P* Value
c-Jun $^{fl/fl}$- IMQ **vs** *c-Jun* $^{\Delta/\Delta}$ *CD11c*-Cre - IMQ	ns	0.2658
**Figure 4C- Mac - *Ccl2***		
Unpaired t test with Welch's correction	Summary	*P* Value

c-Jun $^{fl/fl}$- IMQ **vs** *c-Jun* $^{\Delta/\Delta}$ *CD11c*-Cre - IMQ	ns	0.3438
**Figure 4C- Mac - *Il23p19***		
Unpaired t test	Summary	*P* Value
c-Jun $^{fl/fl}$- IMQ **vs** *c-Jun* $^{\Delta/\Delta}$ *CD11c*-Cre - IMQ	ns	0.7326
**Figure 4E- Mac - *c-Jun***		
Unpaired t test	Summary	Adjusted P Value
c-Jun $^{fl/fl}$- IMQ **vs** *c-Jun* $^{\Delta/\Delta}$ *CD11c*-Cre - IMQ	ns	0.7346
**Figure 4E- Mac - *Ccl2***		
Unpaired t test with Welch's correction	Summary	Adjusted P Value
c-Jun $^{fl/fl}$- IMQ **vs** *c-Jun* $^{\Delta/\Delta}$ *CD11c*-Cre - IMQ	ns	0.307
**Figure 4E- Mac - *Il23p19***		
Unpaired t test	Summary	Adjusted P Value
c-Jun $^{fl/fl}$- IMQ **vs** *c-Jun* $^{\Delta/\Delta}$ *CD11c*-Cre - IMQ	ns	0.4296

Figure 5

**Figure 5B**		
Tukey's multiple comparisons test	Summary	Adjusted *P* Value
c-Jun $^{fl/fl}$ -Veh **vs** c-Jun $^{fl/fl}$ -JNKi	***	0.0009
c-Jun $^{fl/fl}$ -Veh **vs** c-Jun $^{\Delta/\Delta}$ CD11c-Cre	****	<0,0001
c-Jun $^{fl/fl}$ -Veh **vs** Tlr7$^{-/-}$	****	<0,0001
c-Jun $^{\Delta/\Delta}$ CD11c-Cre **vs** c-Jun $^{\Delta/\Delta}$ CD11c-Cre-JNKi	ns	0.7688
Tlr7$^{-/-}$ **vs** Tlr7$^{-/-}$ - JNKi	ns	0.9997
Tlr7$^{-/-}$ **vs** Tlr7$^{-/-}$ c-Jun $^{\Delta/\Delta}$ CD11c-Cre	ns	0.996
**Figure 5C**		
Tukey's multiple comparisons test	Summary	Adjusted *P* Value
c-Jun $^{fl/fl}$ -Veh **vs** c-Jun $^{fl/fl}$ -JNKi	****	<0,0001

c-Jun $^{fl/fl}$ -Veh **vs** c-Jun $^{\Delta/\Delta}$ CD11c-Cre	***	0.0003
c-Jun $^{fl/fl}$ -Veh **vs** Tlr7$^{-/-}$	**	0.004
c-Jun $^{\Delta/\Delta}$ CD11c-Cre **vs** c-Jun $^{\Delta/\Delta}$ CD11c-Cre-JNKi	ns	>0,9999
Tlr7$^{-/-}$ **vs** Tlr7$^{-/-}$ - JNKi	ns	0.9965
Tlr7$^{-/-}$ **vs** Tlr7$^{-/-}$ c-Jun $^{\Delta/\Delta}$ CD11c-Cre	ns	0.9996
**Figure 5D**		
Tukey's multiple comparisons test	Summary	Adjusted *P* Value
c-Jun $^{fl/fl}$ -Veh **vs** c-Jun $^{fl/fl}$ -JNKi	**	0.0022
c-Jun $^{fl/fl}$ -Veh **vs** c-Jun $^{\Delta/\Delta}$ CD11c-Cre	*	0.0363
c-Jun $^{fl/fl}$ -Veh **vs** Tlr7$^{-/-}$	**	0.0034
c-Jun $^{\Delta/\Delta}$ CD11c-Cre **vs** c-Jun $^{\Delta/\Delta}$ CD11c-Cre-JNKi	ns	0.9157
Tlr7$^{-/-}$ **vs** Tlr7$^{-/-}$ - JNKi	ns	>0,9999
Tlr7$^{-/-}$ **vs** Tlr7$^{-/-}$ c-Jun $^{\Delta/\Delta}$ CD11c-Cre	ns	>0,9999
**Figure 5E**		
Tukey's multiple comparisons test	Summary	Adjusted *P* Value
c-Jun $^{fl/fl}$ -Veh **vs** c-Jun $^{fl/fl}$ -JNKi	*	0.0122
c-Jun $^{fl/fl}$ -Veh **vs** c-Jun $^{\Delta/\Delta}$ CD11c-Cre	*	0.0144
c-Jun $^{fl/fl}$ -Veh **vs** Tlr7$^{-/-}$	**	0.0075
c-Jun $^{\Delta/\Delta}$ CD11c-Cre **vs** c-Jun $^{\Delta/\Delta}$ CD11c-Cre-JNKi	ns	0.6378
Tlr7$^{-/-}$ **vs** Tlr7$^{-/-}$ - JNKi	ns	0.6256
Tlr7$^{-/-}$ **vs** Tlr7$^{-/-}$ c-Jun $^{\Delta/\Delta}$ CD11c-Cre	ns	>0,9999
**Figure 5F**		
Tukey's multiple comparisons test	Summary	Adjusted *P* Value
c-Jun $^{fl/fl}$ -Veh **vs** c-Jun $^{fl/fl}$ -JNKi	*	0.0341

c-Jun $^{fl/fl}$ -Veh **vs** c-Jun $^{\Delta/\Delta}$ CD11c-Cre	*	0.0228
c-Jun $^{fl/fl}$ -Veh **vs** Tlr7$^{-/-}$	**	0.0073
c-Jun $^{\Delta/\Delta}$ CD11c-Cre **vs** c-Jun $^{\Delta/\Delta}$ CD11c-Cre-JNKi	ns	0.9975
Tlr7$^{-/-}$ **vs** Tlr7$^{-/-}$ - JNKi	ns	0.9962
Tlr7$^{-/-}$ **vs** Tlr7$^{-/-}$ c-Jun $^{\Delta/\Delta}$ CD11c-Cre	ns	>0,9999
**Figure 5G**		
Tukey's multiple comparisons test	Summary	Adjusted *P* Value
c-Jun $^{fl/fl}$ -Veh **vs** c-Jun $^{fl/fl}$ -JNKi	****	<0,0001
c-Jun $^{fl/fl}$ -Veh **vs** c-Jun $^{\Delta/\Delta}$ CD11c-Cre	****	<0,0001
c-Jun $^{fl/fl}$ -Veh **vs** Tlr7$^{-/-}$	***	0.0002
c-Jun $^{\Delta/\Delta}$ CD11c-Cre **vs** c-Jun $^{\Delta/\Delta}$ CD11c-Cre-JNKi	ns	0.9886
Tlr7$^{-/-}$ **vs** Tlr7$^{-/-}$ - JNKi	ns	0.9998
Tlr7$^{-/-}$ **vs** Tlr7$^{-/-}$ c-Jun $^{\Delta/\Delta}$ CD11c-Cre	ns	>0,999
**Figure 5H**		
Tukey's multiple comparisons test	Summary	Adjusted P Value
c-Jun $^{fl/fl}$ -Veh **vs** c-Jun $^{fl/fl}$ -JNKi	*	0.0151
c-Jun $^{fl/fl}$ -Veh **vs** c-Jun $^{\Delta/\Delta}$ CD11c-Cre	*	0.0189
c-Jun $^{fl/fl}$ -Veh **vs** Tlr7$^{-/-}$	*	0.0473
c-Jun $^{\Delta/\Delta}$ CD11c-Cre **vs** c-Jun $^{\Delta/\Delta}$ CD11c-Cre-JNKi	ns	0.8317
Tlr7$^{-/-}$ **vs** Tlr7$^{-/-}$ - JNKi	ns	>0,9999
Tlr7$^{-/-}$ **vs** Tlr7$^{-/-}$ c-Jun $^{\Delta/\Delta}$ CD11c-Cre	ns	>0,9999
**Figure 5L**		
Paired *t* test	Summary	*P* Value
c-Jun/JunB $^{\Delta/\Delta}$ *K5-Cre-ER*^{T2} Vehicle Day-14 vs Day-28	ns	0.0553

c-Jun/JunB $^{\Delta/\Delta}$ *K5-Cre-ER*^{T2} JNKi Day-14 vs Day-28	*	0.0482
**Figure 5N-Monocytes**		
Bonferroni's multiple comparisons test	Summary	Adjusted P Value
c-Jun/JunB $^{\Delta/\Delta}$ *K5-Cre-ER*^{T2} Vehicle vs JNKi	**	0.0034
**Figure 5N-Neutrophils**		
Bonferroni's multiple comparisons test	Summary	Adjusted P Value
c-Jun/JunB $^{\Delta/\Delta}$ *K5-Cre-ER*^{T2} Vehicle vs JNKi	ns	>0,9999
**Figure 5N-γδ TC**		
Bonferroni's multiple comparisons test	Summary	Adjusted P Value
c-Jun/JunB $^{\Delta/\Delta}$ *K5-Cre-ER*^{T2} Vehicle vs JNKi	*	0.0414

Figure 6

**Figure 6C-CD1a**		
Tukey's multiple comparisons test	Summary	Adjusted *P* Value
Healthy vs Non-Lesional	ns	0.7668
Healthy vs Lesional	***	0.0001
Non-Lesional vs Lesional	***	0.0007
**Figure 6C-CD1c**		
Tukey's multiple comparisons test	Summary	Adjusted *P* Value
Healthy vs Non-Lesional	ns	0.8962
Healthy vs Lesional	*	0.0193
Non-Lesional vs Lesional	ns	0.05
**Figure 6C-CD14**		
Tukey's multiple comparisons test	Summary	Adjusted *P* Value
Healthy vs Non-Lesional	ns	0.2896
Healthy vs Lesional	***	0.001
Non-Lesional vs Lesional	*	0.0333

Figure 7

**Figure 7B - IL-23**		
Paired t test	Summary	*P* Value
R-848 DMSO **vs** R848- JNKi	ns	0.058

Figure 7B - CCL2		
Paired t test	Summary	*P* Value
DMSO **vs** JNKi	ns	0.238
R-848 DMSO **vs** R848- JNKi	*	0.0309
**Figure 7C- IL-23**		
Paired t test	Summary	*P* Value
DMSO **vs** JNKi	ns	0.8837
R-848 DMSO **vs** R848- JNKi	*	0.013
**Figure 7C- CCL2**		
Paired t test	Summary	*P* Value
DMSO **vs** JNKi	ns	0.0501
R-848 DMSO **vs** R848- JNKi	**	0.0012
**Figure 7E- CD80**		
Paired t test	Summary	*P* Value
R-848 DMSO **vs** R848- JNKi	***	0.0006
R-848 DMSO **vs** R848- AP-1 Inh	ns	0.5004
**Figure 7E- CD86**		
Paired t test	Summary	*P* Value
R-848 DMSO **vs** R848- JNKi	**	0.0075
R-848 DMSO **vs** R848- AP-1 Inh.	ns	0.7325
**Figure 7F-CCL2**		
Paired t test	Summary	*P* Value
R-848 DMSO **vs** R848- JNKi	*	0.0161
R-848 DMSO **vs** R848- AP-1 Inh.	*	0.0342
**Figure 7F- IL-23**		
Paired t test	Summary	*P* Value
R-848 DMSO **vs** R848- JNKi	*	0.0154
R-848 DMSO **vs** R848- AP-1 Inh.	*	0.0138

EV Figure 1

Figure EV1E		
Tukey's multiple comparisons test	Summary	Adjusted *P* Value
c-Jun $^{fl/fl}$ ***vs*** *c-Jun* $^{fl/fl}$ *36h*	***	0.0004
c-Jun $^{fl/fl}$ ***vs*** *c-Jun* $^{fl/fl}$ *48h*	****	<0,0001
c-Jun $^{fl/fl}$ ***vs*** *c-Jun* $^{\Delta/\Delta}$ *CD11c-Cre 72h*	****	<0,0001
c-Jun $^{fl/fl}$ *36h* ***vs*** *c-Jun* $^{\Delta/\Delta}$ *CD11c-Cre 36h*	**	0.0011
c-Jun $^{fl/fl}$ *48h* ***vs*** *c-Jun* $^{\Delta/\Delta}$ *CD11c-Cre 48h*	***	0.0001
c-Jun $^{fl/fl}$ *72h* ***vs*** *c-Jun* $^{\Delta/\Delta}$ *CD11c-Cre 72h*	****	<0,0001

Figure EV1F		
Tukey's multiple comparisons test	Summary	Adjusted *P* Value
c-Jun $^{fl/fl}$ ***vs*** *c-Jun* $^{fl/fl}$ *36h*	***	0.0004
c-Jun $^{fl/fl}$ ***vs*** *c-Jun* $^{fl/fl}$ *48h*	****	<0,0001
c-Jun $^{fl/fl}$ ***vs*** *c-Jun* $^{fl/fl}$ *72h*	***	0.0003
c-Jun $^{fl/fl}$ *36h* ***vs*** *c-Jun* $^{\Delta/\Delta}$ *CD11c-Cre 36h*	*	0.0322
c-Jun $^{fl/fl}$ *48h* ***vs*** *c-Jun* $^{\Delta/\Delta}$ *CD11c-Cre 48h*	****	<0,0001

Figure EV1G		
Tukey's multiple comparisons test	Summary	Adjusted *P* Value
c-Jun $^{fl/fl}$ ***vs*** *c-Jun* $^{fl/fl}$ 2 days	*	0.0491
c-Jun $^{fl/fl}$ ***vs*** *c-Jun* $^{fl/fl}$ 3 days	*	0.0438
c-Jun $^{fl/fl}$ ***vs*** *c-Jun* $^{fl/fl}$ 5 days	****	<0,0001

Figure EV1H		
Tukey's multiple comparisons test	Summary	Adjusted *P* Value
c-Jun $^{fl/fl}$ ***vs*** *c-Jun* $^{fl/fl}$ 5 days	ns	0.072

Figure EV1I		
Tukey's multiple comparisons test	Summary	Adjusted *P* Value
c-Jun $^{fl/fl}$ ***vs*** *c-Jun* $^{fl/fl}$ 2 days	*	0.0437
c-Jun $^{fl/fl}$ ***vs*** *c-Jun* $^{fl/fl}$ 3 days	**	0.009
c-Jun $^{fl/fl}$ ***vs*** *c-Jun* $^{fl/fl}$ 5 days	*	0.0101

EV Figure 2

Figure EV2B-Cxcl1		
Unpaired t test with Welch's correction	Summary	P Value
c-Jun $^{fl/fl}$-32h **vs** c-Jun $^{\Delta/\Delta}$ CD11c-Cre-32h *	ns	0.4137
c-Jun $^{fl/fl}$-48h **vs** c-Jun $^{\Delta/\Delta}$ CD11c-Cre-48h	***	0.001
**Figure EV2B-Ccl20**		
Unpaired t test	Summary	P Value
c-Jun $^{fl/fl}$-32h **vs** c-Jun $^{\Delta/\Delta}$ CD11c-Cre-32h *	ns	0.1655
c-Jun $^{fl/fl}$-48h **vs** c-Jun $^{\Delta/\Delta}$ CD11c-Cre-48h	*	0.0376
**Figure EV2B-S100a8**		
Unpaired t test with Welch's correction	Summary	P Value
c-Jun $^{fl/fl}$-32h **vs** c-Jun $^{\Delta/\Delta}$ CD11c-Cre-32h	ns	0.4487
c-Jun $^{fl/fl}$-48h **vs** c-Jun $^{\Delta/\Delta}$ CD11c-Cre-48h	**	0.0023
**Figure EV2B-S100a9**		
Unpaired t test with Welch's correction	Summary	P Value
c-Jun $^{fl/fl}$-32h **vs** c-Jun $^{\Delta/\Delta}$ CD11c-Cre-32h *	ns	0.6055
c-Jun $^{fl/fl}$-48h **vs** c-Jun $^{\Delta/\Delta}$ CD11c-Cre-48h	ns	0.0766

**Figure EV2B-*Il22***		
Unpaired t test with Welch's correction	Summary	P Value
c-Jun $^{fl/fl}$-32h **vs**<br>c-Jun $^{\Delta/\Delta}$ CD11c-Cre-32h *	ns	0.9803
c-Jun $^{fl/fl}$-48h **vs**<br>c-Jun $^{\Delta/\Delta}$ CD11c-Cre-48h	*	0.0102

**Figure EV2B-*Il6***		
Unpaired t test with Welch's correction	Summary	P Value
c-Jun $^{fl/fl}$-32h **vs**<br>c-Jun $^{\Delta/\Delta}$ CD11c-Cre-32h *	ns	0.6252
c-Jun $^{fl/fl}$-48h **vs**<br>c-Jun $^{\Delta/\Delta}$ CD11c-Cre-48h	*	0.0142

**Figure EV2B-*Tnfa***		
Unpaired t test	Summary	P Value
c-Jun $^{fl/fl}$-32h **vs**<br>c-Jun $^{\Delta/\Delta}$ CD11c-Cre-32h	ns	0.1399
c-Jun $^{fl/fl}$-48h **vs**<br>c-Jun $^{\Delta/\Delta}$ CD11c-Cre-48h	*	0.0275

**Figure EV2B-*Il1b***		
Unpaired t test	Summary	P Value
c-Jun $^{fl/fl}$-32h **vs**<br>c-Jun $^{\Delta/\Delta}$ CD11c-Cre-32h	ns	0.7375
c-Jun $^{fl/fl}$-48h **vs**<br>c-Jun $^{\Delta/\Delta}$ CD11c-Cre-48h	ns	0.1058

**Figure EV2C**		
Unpaired t test with Welch's correction	Summary	P Value
c-Jun $^{fl/fl}$ - IMQ **vs**<br>c-Jun $^{\Delta/\Delta}$ Mx1-Cre - IMQ - ***Il22***	*	0.0439
Unpaired t test	Summary	P Value
c-Jun $^{fl/fl}$ - IMQ **vs**<br>c-Jun $^{\Delta/\Delta}$ Mx1-Cre - IMQ - ***Il23p19***	*	0.0145
c-Jun $^{fl/fl}$ - IMQ **vs**<br>c-Jun $^{\Delta/\Delta}$ Mx1-Cre - IMQ - ***Il6***	*	0.0155
c-Jun $^{fl/fl}$ - IMQ **vs**<br>c-Jun $^{\Delta/\Delta}$ Mx1-Cre - IMQ - ***Ccl20***	**	0.0025
c-Jun $^{fl/fl}$ - IMQ **vs**<br>c-Jun $^{\Delta/\Delta}$ Mx1-Cre - IMQ - ***Cxcl1***	*	0.0206
c-Jun $^{fl/fl}$ - IMQ **vs**<br>c-Jun $^{\Delta/\Delta}$ Mx1-Cre - IMQ - ***S100a8***	ns	0.7598
c-Jun $^{fl/fl}$ - IMQ **vs**<br>c-Jun $^{\Delta/\Delta}$ Mx1-Cre - IMQ - ***S100a9***	ns	0.638

c-Jun $^{fl/fl}$ - IMQ **vs**<br>c-Jun $^{\Delta/\Delta}$ Mx1-Cre - IMQ - ***Il1b***	ns	0.2366
c-Jun $^{fl/fl}$ - IMQ **vs**<br>c-Jun $^{\Delta/\Delta}$ Mx1-Cre - IMQ - ***Tnfa***	ns	0.135
**Figure EV2F**		
Unpaired t test	Summary	P Value
Wild-type- **vs**<br>Wild-type - IMQ - **CD45-**	**	0.0047
Wild-type- **vs**<br>Wild-type - IMQ - **Gr-1**	ns	0.2262
**Figure EV2H**		
Tukey's multiple comparisons test	Summary	Adjusted P Value
c-Jun $^{fl/fl}$ - LAL **vs**<br>c-Jun $^{fl/fl}$ - IMQ	****	<0,0001
c-Jun $^{fl/fl}$ -IMQ **vs**<br>c-Jun $^{\Delta/\Delta}$ Mx1-Cre - IMQ	ns	0.3345
**Figure EV2I**		
Tukey's multiple comparisons test	Summary	Adjusted P Value
IMQ **vs**<br>IMQ-NF-κBi	***	0.0003

EV Figure 3

**Figure EV3H**		
Tukey's multiple comparisons test	Summary	Adjusted P Value
Wild-type<br>mouse IgG1 vs JNKi	*	0.0269
Wild-type<br>mouse IgG1 vs αIL23R	*	0.0245
Wild-type<br>JNKi vs αIL23R	ns	0.9963
**Figure EV3I-Monocytes**		
Tukey's multiple comparisons test	Summary	Adjusted P Value
Wild-type<br>mouse IgG1 vs JNKi	ns	0.0576
Wild-type<br>mouse IgG1 vs αIL23R	ns	0.0576
Wild-type<br>JNKi vs αIL23R	ns	>0,9999
**Figure EV3I-Neutrophils**		
Tukey's multiple comparisons test	Summary	Adjusted P Value
Wild-type<br>mouse IgG1 vs JNKi	***	0.0006

Wild-type<br>mouse IgG1 vs αIL23R	**	0.0016
Wild-type<br>JNKi vs αIL23R	ns	0.4827
**Figure EV3I-γδ TC**		
Tukey's multiple comparisons test	Summary	Adjusted P Value
Wild-type<br>mouse IgG1 vs JNKi	ns	0.077
Wild-type<br>mouse IgG1 vs αIL23R	*	0.0311
Wild-type<br>JNKi vs αIL23R	ns	0.7504

EV Figure 5

**FigureEV5B- CD80**		
Paired t test	Summary	P Value
DMSO **vs**<br>- JNKi	ns	0.0543
R-848 DMSO **vs**<br>R848- JNKi	ns	0.0847
**FigureEV5B- CD86**		
Paired t test	Summary	P Value
DMSO **vs**<br>- JNKi	ns	0.2091
R-848 DMSO **vs**<br>R848- JNKi	ns	0.3187
**FigureEV5D- CD80**		
Paired t test	Summary	P Value
DMSO **vs**<br>JNKi	ns	0.1275
R-848 DMSO **vs**<br>R848- JNKi	ns	0.843
**FigureEV5D- CD86**		
Paired t test	Summary	P Value
DMSO **vs**<br>JNKi	*	0.0448
R-848 DMSO **vs**<br>R848- JNKi	ns	0.9272
**FigureEV5F- CD80**		
Tukey's multiple comparisons test	Summary	Adjusted P Value
Untreated **vs**<br>LL-37-RNA-40	*	0.0104
Untreated **vs**<br>RNA-40	ns	0.1643

Untreated **vs** LL-37	ns	0.2922
**FigureEV5F- CD86**		
Tukey's multiple comparisons test	Summary	Adjusted P Value
Untreated **vs** LL-37-RNA-40	**	0.0011
Untreated **vs** RNA-40	ns	0.5081
Untreated **vs** LL-37	ns	0.872
**FigureEV5G- IL-23**		
Tukey's multiple comparisons test	Summary	Adjusted P Value
Untreated **vs** LL-37-RNA-40	***	0.0002
Untreated **vs** RNA-40	ns	>0,9999
Untreated **vs** LL-37	ns	0.9998
**FigureEV5G- CCL2**		
Tukey's multiple comparisons test	Summary	Adjusted P Value
Untreated **vs** LL-37-RNA-40	**	0.0014
Untreated **vs** RNA-40	ns	0.9997
Untreated **vs** LL-37	ns	0.9997

Appendix Figure S2

**Appendix Figure S2B**		
Tukey's multiple comparisons test	Summary	Adjusted P Value
Wild-type-1.5 days **vs** CCL2 $^{-/-}$ - 1.5 days	ns	0.9937
Wild-type-3 days **vs** CCL2 $^{-/-}$ - 3 days	ns	>0,9999
**Appendix Figure S2C- TEWL**		
2 way - ANOVA	Summary	*P* Value
Wild-type- vs *Ccl2* $^{-/-}$	ns	0.7566
**Appendix Figure S2E- Monocytes**		
Unpaired *t*-test	Summary	*P* Value
Wild-type- IMQ vs *Ccl2* $^{-/-}$ + IMQ	*	0.0123
**Appendix Figure S2E- gd T cells**		
Unpaired *t*-test	Summary	*P* Value
Wild-type- IMQ vs *Ccl2* $^{-/-}$ + IMQ	ns	0.7771
**Appendix Figure S2E- Macrophages**		
Unpaired *t*-test	Summary	*P* Value
Wild-type- IMQ vs *Ccl2* $^{-/-}$ + IMQ	**	0.0033
**Appendix Figure S2E- Neutrophils**		
Unpaired *t*-test	Summary	*P* Value
Wild-type- IMQ vs *Ccl2* $^{-/-}$ + IMQ	ns	0.289

Appendix Figure S3

**Appendix Figure S3A**		
One way - ANOVA -Tukey	Summary	*P* Value
Tlr7 $^{-/-}$ *LAL* vs *Tlr7* $^{-/-}$ AP-1 Inh.	*	0.0379
Tlr7 $^{-/-}$ *LAL* vs *Tlr7* $^{-/-}$ AP-1 Inh. + IMQ	ns	0.0885
c-Jun $^{fl/fl}$ *LAL* vs *c-Jun* $^{fl/fl}$ AP-1 Inh.	***	0.0001
c-Jun $^{fl/fl}$ *LAL* vs *c-Jun* $^{fl/fl}$ + IMQ	**	0.0017
c-Jun $^{fl/fl}$ *LAL* vs *c-Jun* $^{fl/fl}$ AP-1 Inh. + IMQ	****	<0.0001
c-Jun $^{fl/fl}$ *IMQ* vs *c-Jun* $^{fl/fl}$ AP-1 Inh. + IMQ	*	0.0293
c-Jun $^{\Delta/\Delta}$ *Mx1-Cre* LAL vs AP-1 Inhibitor	*	0.0292
c-Jun $^{\Delta/\Delta}$ *Mx1-Cre* LAL vs AP-1 Inhibitor + IMQ	***	0.0005

**Appendix Figure S3B**		
One way - ANOVA - Tukey	Summary	*P* Value
Tlr7 $^{-/-}$ *LAL* vs *Tlr7* $^{-/-}$ AP-1 Inh.	*	0.0452
Tlr7 $^{-/-}$ *LAL* vs *Tlr7* $^{-/-}$ AP-1 Inh. + IMQ	ns	0.09
c-Jun $^{fl/fl}$ *LAL* vs *c-Jun* $^{fl/fl}$ AP-1 Inh.	**	0.0011
c-Jun $^{fl/fl}$ *LAL* vs *c-Jun* $^{fl/fl}$ + IMQ	ns	0.05
c-Jun $^{fl/fl}$ *LAL* vs *c-Jun* $^{fl/fl}$ AP-1 Inh. + IMQ	****	< 0.0001
c-Jun $^{\Delta/\Delta}$ *Mx1-Cre* LAL vs AP-1 Inhibitor + IMQ	**	0.0037

**Appendix Figure S3C**		
One way - ANOVA - Tukey	Summary	*P* Value
c-Jun $^{fl/fl}$ *LAL* vs *c-Jun* $^{fl/fl}$ + IMQ	****	< 0.0001
c-Jun $^{fl/fl}$ *LAL* vs *c-Jun* $^{fl/fl}$ AP-1 Inh. + IMQ	**	0.0011
c-Jun $^{fl/fl}$ IMQ vs c-Jun $^{fl/fl}$ AP-1 Inhibitor IMQ	****	< 0.0001

**Appendix Figure S3D**		
One way - ANOVA - Tukey	Summary	*P* Value
c-Jun $^{fl/fl}$ *LAL* vs<br>*c-Jun* $^{fl/fl}$ + IMQ	****	< 0.0001
c-Jun $^{fl/fl}$ *IMQ* vs<br>*c-Jun* $^{fl/fl}$ AP-1 Inh. + IMQ	****	< 0.0001
c-Jun $^{\Delta/\Delta}$ *Mx1-Cre*<br>LAL vs IMQ	****	< 0.0001

Appendix Figure S4

**Appendix Figure S4B**		
One way - ANOVA Dunnett	Summary	*P* Value
Il17a : PBS vs 12h	**	0.0024
Il17a : PBS vs 36h	****	< 0.0001
Ccl2 : PBS vs 12h	*	0.0318
**Appendix Figure S4C**		
Welch´s *t*-test	Summary	*P* Value
Il-12rb1 : LAL **vs** IMQ	*	0.0114
Il-12rb2 : LAL **vs** IMQ	**	0.0049
**Appendix Figure S4D**		
One way - ANOVA - Tukey	Summary	*P* Value
Gzmb: LAL **vs** rIL-12	****	< 0.0001
Trail: LAL **vs** rIL-12	*	0.0276
Ifng: LAL **vs** rIL-12	*	0.0403

Appendix Figure S5

**Appendix Figure S5A**		
One way - ANOVA Tukey	Summary	*P* Value
c-Jun $^{fl/fl}$ LAL **vs**<br>c-Jun$^{fl/fl}$ CL264	***	0.0001
c-Jun $^{fl/fl}$ LAL **vs**<br>c-Jun$^{fl/fl}$ CL307	**	0.0018
c-Jun $^{\Delta/\Delta}$ *Mx1-Cre*<br>LAL vs CL264	*	0.0261
c-Jun $^{\Delta/\Delta}$ *Mx1-Cre*<br>LAL vs CL307	*	0.0441
**Appendix Figure S5B**		
One way - ANOVA Tukey	Summary	*P* Value
c-Jun $^{fl/fl}$ LAL **vs**<br>c-Jun$^{fl/fl}$ CL264	*	0.0411
c-Jun $^{fl/fl}$ LAL **vs**<br>c-Jun$^{fl/fl}$ CL307	**	0.0053

Appendix Figure S5C		
One way - ANOVA Tukey	Summary	*P* Value
c-Jun$^{fl/fl}$ LAL **vs** c-Jun$^{fl/fl}$ CL264	****	< 0.0001
c-Jun$^{fl/fl}$ LAL **vs** c-Jun$^{fl/fl}$ CL307	****	< 0.0001
CL264*: c-Jun*$^{fl/fl}$ **vs** c-Jun $^{Δ/Δ}$ Mx1-Cre	***	0.0002
CL307*: c-Jun*$^{fl/fl}$ **vs** c-Jun $^{Δ/Δ}$ Mx1-Cre	****	< 0.0001

Appendix Figure S5D		
One way - ANOVA Tukey	Summary	*P* Value
c-Jun$^{fl/fl}$ LAL **vs** c-Jun$^{fl/fl}$ CL264	****	< 0.0001
c-Jun$^{fl/fl}$ LAL **vs** c-Jun$^{fl/fl}$ CL307	****	< 0.0001
CL264*: c-Jun*$^{fl/fl}$ **vs** c-Jun $^{Δ/Δ}$ Mx1-Cre	**	0.0034
CL307*: c-Jun*$^{fl/fl}$ **vs** c-Jun $^{Δ/Δ}$ Mx1-Cre	***	0.0006
c-Jun $^{Δ/Δ}$ *Mx1-Cre* LAL vs CL264	****	< 0.0001
c-Jun $^{Δ/Δ}$ *Mx1-Cre* LAL vs CL307	****	< 0.0001

Appendix Figure S6

Appendix Figure S6B		
One way - ANOVA Tukey	Summary	*P* Value
Gr-1⁺ vs. CD45⁻	*	*0.0137*
Gr-1⁺ vs. CD3ε⁺	**	*0.0097*
CD45⁻ vs. CD3ε⁺	*ns*	*0.9998*

Appendix Figure S7

Appendix Figure S7C		
One way - ANOVA Tukey	Summary	*P* Value
CCL2: IMQ-Vehicle **vs** IMQ- JNKi	**	0.0016
CXCL1: IMQ-Vehicle **vs** IMQ- JNKi	**	0.002
TNFα: IMQ-Vehicle **vs** IMQ- JNKi	ns	0.3157
TSLP: IMQ-Vehicle **vs** IMQ- JNKi	*	0.0118
IL-17A: IMQ-Vehicle **vs** IMQ- JNKi	****	<0,0001
IL-18: IMQ-Vehicle **vs** IMQ- JNKi	ns	0.128
IL-33: IMQ-Vehicle **vs** IMQ- JNKi	*	0.0485
