# Supplementary material for: Psoriatic skin inflammation is promoted by c‐Jun/AP‐1‐dependent CCL2 and IL‐23 expression in dendritic cells
Source: EMBO Mol Med. 2021 Mar 16;13(4):e12409. doi: 10.15252/emmm.202012409 (PMC8033525; doi:10.15252/emmm.202012409)
Supplement: Supplementary file 2 — Expanded View Figures PDF [file EMMM-13-e12409-s001.pdf]

Expanded View Figures

Figure EV1. c-Jun in DCs is required for IMQ-induced keratinocyte proliferation and differentiation, but is dispensable for skin DC development.

A Graphical timeline of IMQ treatment for the different Cre-Lines. Mice received poly I:C (*Mx1*-Cre) or Tamoxifen (*K5*-Cre-ER^{T2}) for cell-type specific deletion of c-Jun and were waxed 2 days before start of IMQ treatment. IMQ was applied daily on the back skin and mice were taken for analysis after 1.5, 3, or 7 days after the first IMQ application, which corresponds to an IMQ treatment of 1, 3 or 7 times, respectively. After 7 days of IMQ application, mice were left untreated for another 3 days to study the resolution of IMQ-induced skin inflammation. Controls (*c-Jun*$^{fl/fl}$) were treated equally to *Mx-1* or *K5*-Cre-ER^{T2} mice in all experiments, which is indicated by a color code (bronze = poly I:C and green = Tamoxifen) here and for all Figures henceforth.

B–D Validation of the deletion efficiency of c-Jun in the *Mx1*-Cre, the *CD11c*-Cre and *K5*-Cre-ER^{T2} mouse model. Immunofluorescence of c-Jun (green), K5 (red) and CD45 (white) (B), of c-Jun (green) and CD11c (red) (C) and of c-Jun (green) and K5 (red) (D) was performed in back skin from the indicated genotypes. DAPI was used to counterstain. Arrows indicate CD45^{+} (B) or CD11c^{+} skin cells (C). Magnification: 25× (B), 40× (C), 40× (D). Scale Bar: 50 μm.

E, F Ratio of K5 to K10 positive areas (E), and number of Ki-67^{+} cells (F) in epidermis of indicated mice was analyzed by immunofluorescence performed as described in Appendix Fig S1A and B. Three randomly chosen fields per section were analyzed for each sample (n = 6–10 (E), n = 5–12 (F); 2–4 independent experiments)

G–I Flow cytometry of DC subsets in total skin of indicated mice after 2, 3 and 5d of daily IMQ treatment. CD11b^{+} DC cells were defined as MHCII^{+}CD172^{+}CD11b^{+}CD64^{-} (G), CD103^{+} DC as MHCII^{+}XCR1^{+}CD103^{+}CD64^{-} (H) and Langerhans cells as MHCII^{+}CD172^{+}CD207^{+} CD64^{-} (I) among live, single, CD45^{+} cells. Graphs show DC subsets as % of live, single cells (n = 6–12 (G), n = 4–11 (H), n = 5–13 (I); 2–4 independent experiments).

Data information: Data are shown as mean ± SEM. P-values were calculated by one-way ANOVA with Tukey (E, F) or Bonferroni multiple comparison test (G-I). Statistical significance: ns > 0.05, *P < 0.05, **P < 0.01, ***P < 0.001, ****P < 0.0001. See Appendix Table S3 for exact P-values.

Source data are available online for this figure.

© 2021 The Authors

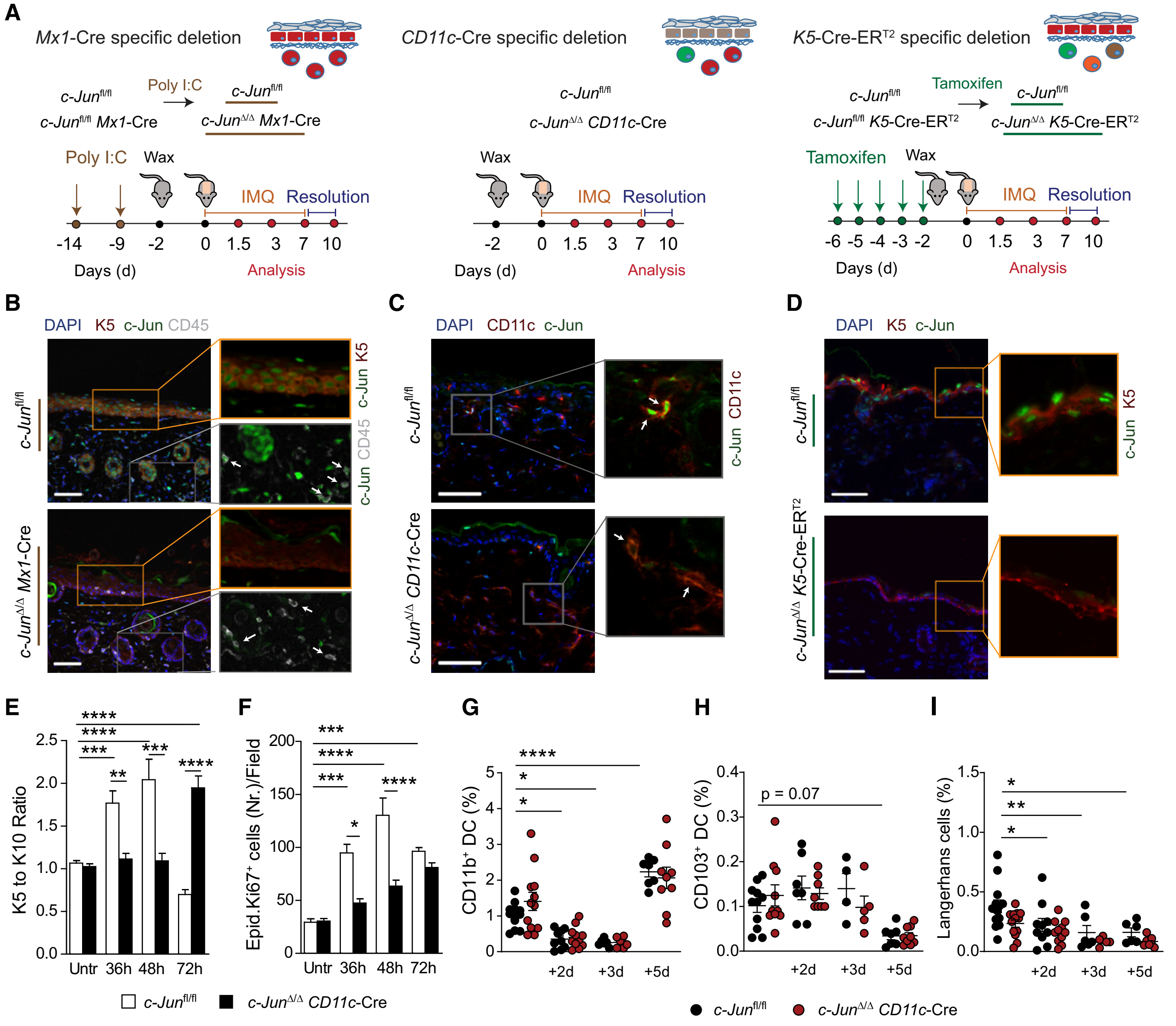

Figure EV1.

 © 2021 The Authors

Figure EV2. Control of cytokine expression in IMQ-treated skin and BMDCs by c-Jun.

A Immunofluorescence of c-Jun (green), CD11c (red) and DAPI in mouse back skin of indicated mice treated with IMQ for 8 h. Insets give an enlarged view of the framed area. Scale bar: 10 µm, Magnification: 40×.

B qRT–PCR detection of chemokines *Cxcl1*, *Ccl20*, antimicrobial proteins *S100a8*, *S100a9* and the pro-inflammatory cytokines *Tnfa*, *Il22*, *Il6* and *Il1b* in total RNA isolated from the back skin of indicated mice treated with IMQ for 32 or 48 h (n = 9–20; 3–6 experiments).

C qRT–PCR mRNA expression analysis of indicated cytokines in BMDCs. Bar graph shows fold change of targets in IMQ (4 h) stimulated BMDCs relative to LAL. Asterisk shows significance between the genotypes in IMQ-treated samples (n = 3–7; 2–3 independent experiments).

D Immunofluorescence of CD11c (green), IL-23p19 (red) and DAPI in indicated BMDCs stimulated with IMQ for 5 h in the presence of Brefeldin A. Scale bar: 25 µm, Magnification: 60×.

E Gating strategy to sort DCs ($CD45^{+}CD11c^{+}$ $MHCII^{+}$), T cells ($CD3\varepsilon^{+}$), granulocytes ($Gr\text{-}1^{+}$) and non-immune cells ($CD45^{-}$) from back skin treated with IMQ for 8 h.

F qRT–PCR detection of *Il23p19* mRNA expression levels in $CD3\varepsilon^{+}$, $Gr\text{-}1^{+}$ and $CD45^{-}$ cells sorted from back skin as described in (E) (n = 4–6, 3 independent experiments).

G IL-23p19 and IL-12p40 expression in BMDCs was analyzed by intracellular flow cytometry. BMDCs were stimulated with IMQ for 4 h in the presence of Brefeldin A. Representative plots shown are pregated on live, single, $CD45^{+}$ and $CD11c^{+}$ cells.

H Bar graph shows % of live, single, $CD45^{+}$, $CD11c^{+}$ cells (% DCs) expressing IL12-p40 (n = 7–10; 3 independent experiments).

I qRT–PCR detection of *Il23p19* mRNA expression in BMDCs pretreated with MG-132 (NF-κBi, 20 µM) for 1 h and stimulated with IMQ for 4 h (n = 5–6; 2 independent experiments).

Data information: Data are shown as mean ± SEM. P-values were calculated by unpaired, two-tailed t-test (B, C, and F) or one-way ANOVA with Tukey multiple comparison test (H, I). Statistical significance: ns > 0.05, *$P < 0.05$, **$P < 0.01$, ***$P < 0.001$, ****$P < 0.0001$. See Appendix Table S3 for exact P-values.

Source data are available online for this figure.

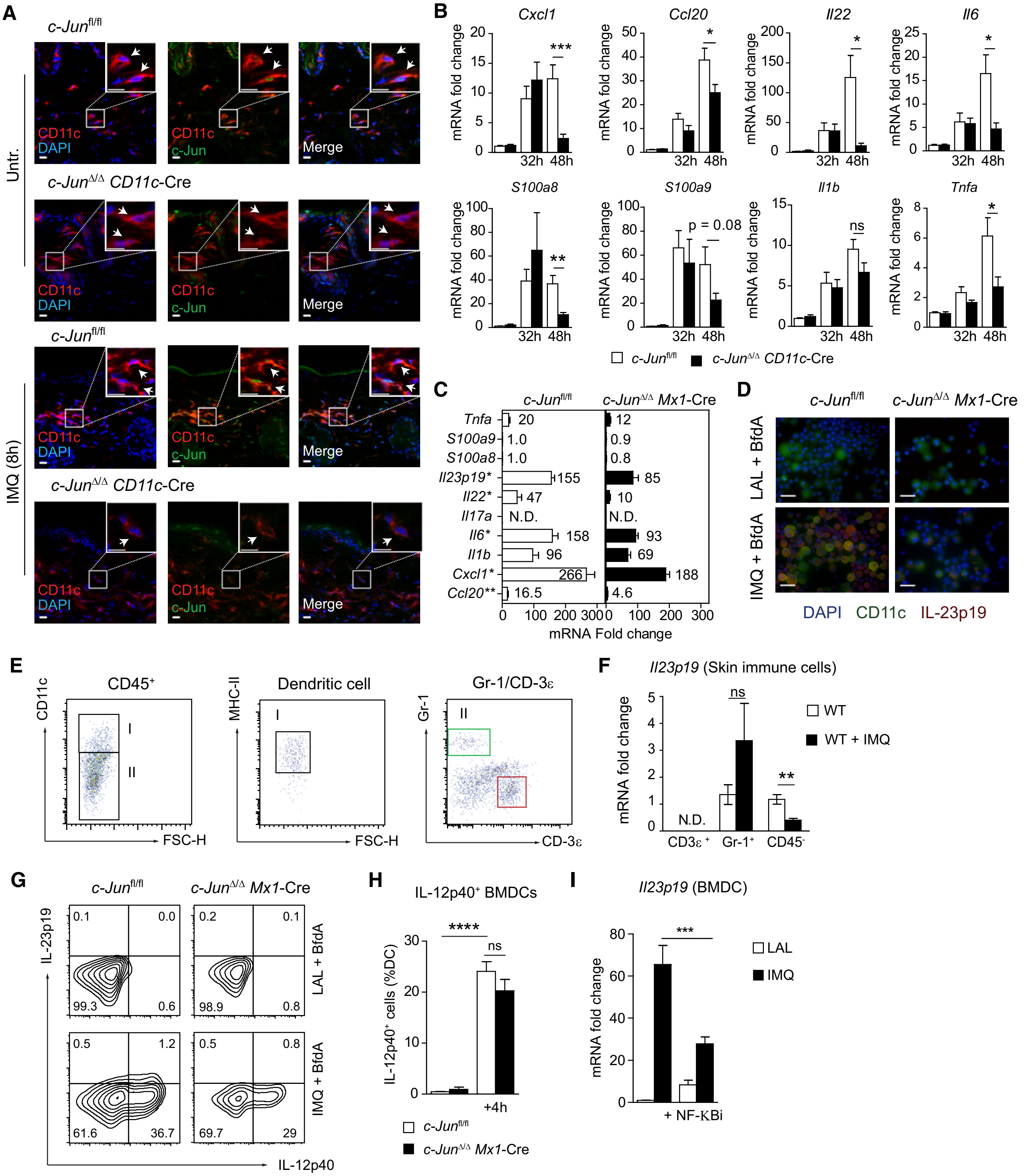

Figure EV2.

© 2021 The Authors

Figure EV3. Validation of the therapeutic potential of JNK inhibition in psoriasis-like mouse models.

A–C Back skin of *c-Jun*$^{fl/fl}$, *c-Jun*$^{\Delta/\Delta}$*CD11c*-Cre, *Tlr7*$^{-/-}$ and *Tlr7*$^{-/-}$ *c-Jun*$^{\Delta/\Delta}$*CD11c*-Cre mice was treated with IMQ and JNKi or vehicle for 5 days. Shown are representative H&E stained sections (Bright-field images, Magnification: 20×, Scale bar: 100 μm) (A), immunofluorescence staining's of Ki-67 (green) and DAPI (Scale bar: 100 μm, Magnification: 20×) (B) and of K5 (red), K10 (green) and DAPI (Scale bar: 100 μm, Magnification: 20×) (C) in murine back skin. For quantification 2–3 randomly chosen fields per section were counted.

D–F Flow cytometric analysis of cell suspension from back skin of indicated mice and treatment. t-SNE- plots (D) show the cutaneous immune cell phenotype. Populations defined by t-SNE algorithm were confirmed by conventional gating as described in Appendix Fig S7A and representative plots are shown in (E) for Monocytes and Neutrophils and in (F) for γδ T cells and dendritic epidermal T cells (DETC). Numbers adjacent to marked areas indicate percentage of cell population among CD45^{+} cells (D) and live, single cells (E, F). Representative plots shown are pregated on live, single, CD45^{+} (D) and CD11b^{+} (E) or CD11b^{-} cells (F).

G Experimental design for blocking IL-23 signaling in the IMQ-induced skin inflammation model. Back skin of wild-type mice was treated daily with IMQ and anti-IL23R antibody (15 mg/kg, i.p.) or isotype control for 5 consecutive days. To compare JNK Inhibitor (15 mg/kg, i.p.) was given (n = 3)

H Trans-epidermal water loss (TEWL) was analyzed in the back skin at the end of treatment.

I Flow cytometry of total back skin after 5 days of IMQ treatment. Analyzed were dermal γδ T cells (γδ TCR^{int+}), Monocytes (CD11b^{+}Ly6C^{hi}), and Neutrophils (CD11b^{+}Ly6G^{+}). Graphs show immune cells as % of live, single cells.

Data information: Data are shown as mean ± SEM. P-values were calculated by Tukey multiple comparison test (H, I). Statistical significance: ns > 0.05, *P < 0.05, **P < 0.01, ***P < 0.001. See Appendix Table S3 for exact P-values.

Source data are available online for this figure.

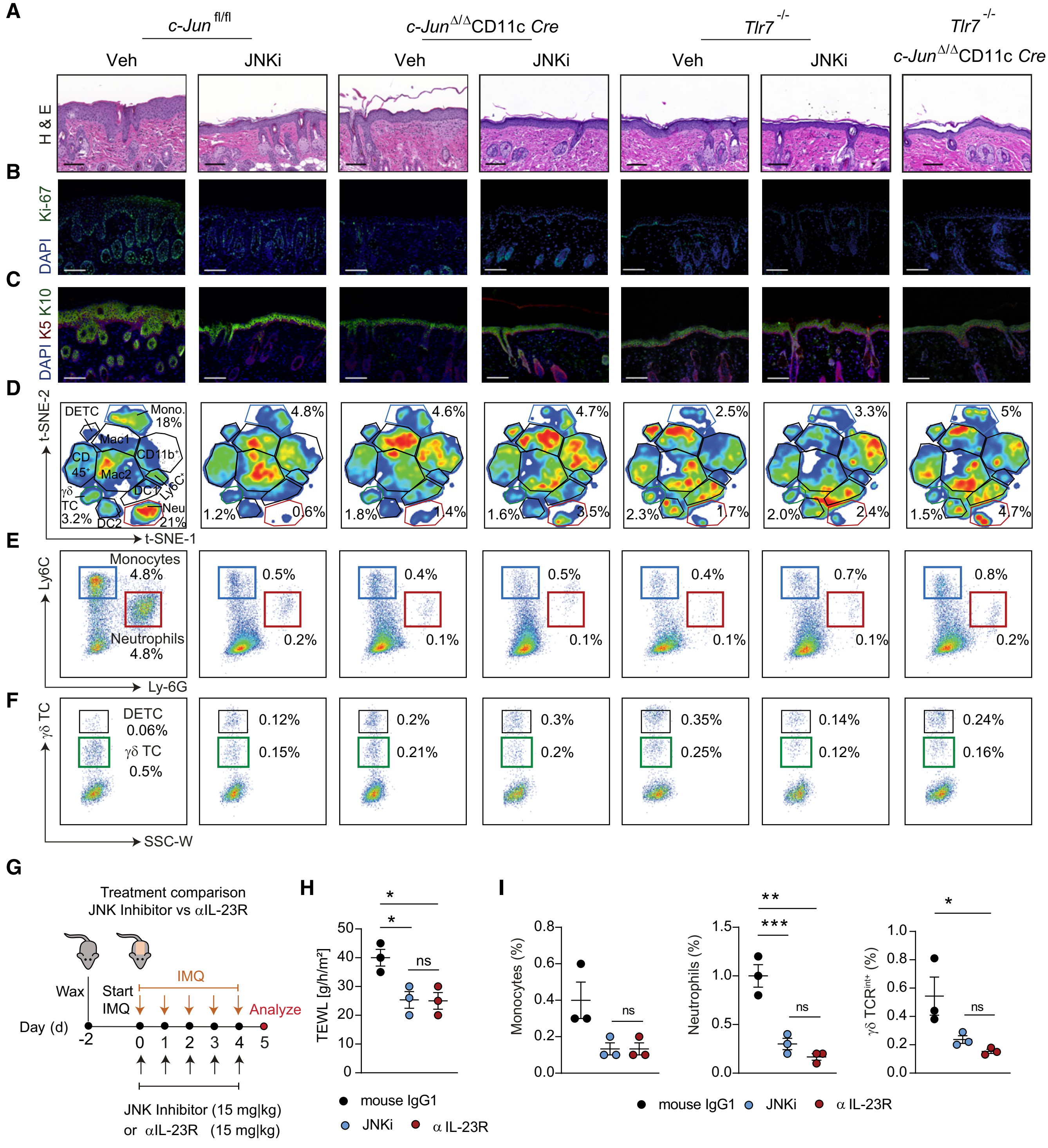

Figure EV3.

 © 2021 The Authors

Figure EV4. Characterization of c-Jun co-expression with CCL2 and IL-23 in DCs of lesional psoriatic skin.

A Immunofluorescence of c-Jun (green), CD11c (red) and DAPI in psoriatic lesions from two patients and in non-lesional skin from one patient. Insets give an enlarged view of the framed area. Arrows indicate $CD11c^+$ skin cells. Scale bar: 10 μm, Magnification: 40x.

B Linear regression analysis between *c-Jun* and *Ccl2* in healthy (n = 38), non-lesional (n = 27) and lesional (n = 28) skin. Expression values were obtained from the GEO Data Set [GSE121212]. A Pearson's correlation test was performed and r^2 and P values are indicated in the plot.

C Expression values of *Tlr7*, *Tlr8*, *Jnk1, Jnk2, Jnk3, c-Jun, Ccl2 and Il23p19* in healthy (n = 38), non-lesional (n = 27) and lesional (n = 28) skin. Expression values were obtained from the GEO Data Set [GSE121212]. Box and whiskers plot: Central band shows median, box extends from the 25th to 75th percentiles and whiskers go down to the smallest (min) and up to the largest (max) value.

D Representative immunofluorescence of CCL2 or IL-23p19 (green) and DAPI in healthy and lesional skin. Arrows indicate $IL\text{-}23p19^+$ (upper panels) or $CCL2^+$ dermal cells (lower panels). Magnification: 40x. Scale bar: 50 μm (n = 2 patient samples).

E, F Representative immunofluorescence of CCL2 (E) or IL-23p19 (F) (white), c-Jun (green) and the DC markers CD1c or CD14 (red) was performed on lesional psoriatic skin. DAPI was used to counterstain. Arrows indicate triple-positive cells and an asterisk highlights a representative one that is shown enlarged in a white-framed inlet. Magnification: 40×. Scale bar: 50 μm (n = 2 patient samples).

Data information: Data are shown as mean ± SEM. P-values were calculated by Pearson´s correlation test (B) and one-way ANOVA with Tukey multiple comparison test (C). Statistical significance: ns > 0.05, $^{**}P < 0.01$, $^{***}P < 0.001$, $^{****}P < 0.0001$. See Appendix Table S3 for exact P-values.

Source data are available online for this figure.

© 2021 The Authors

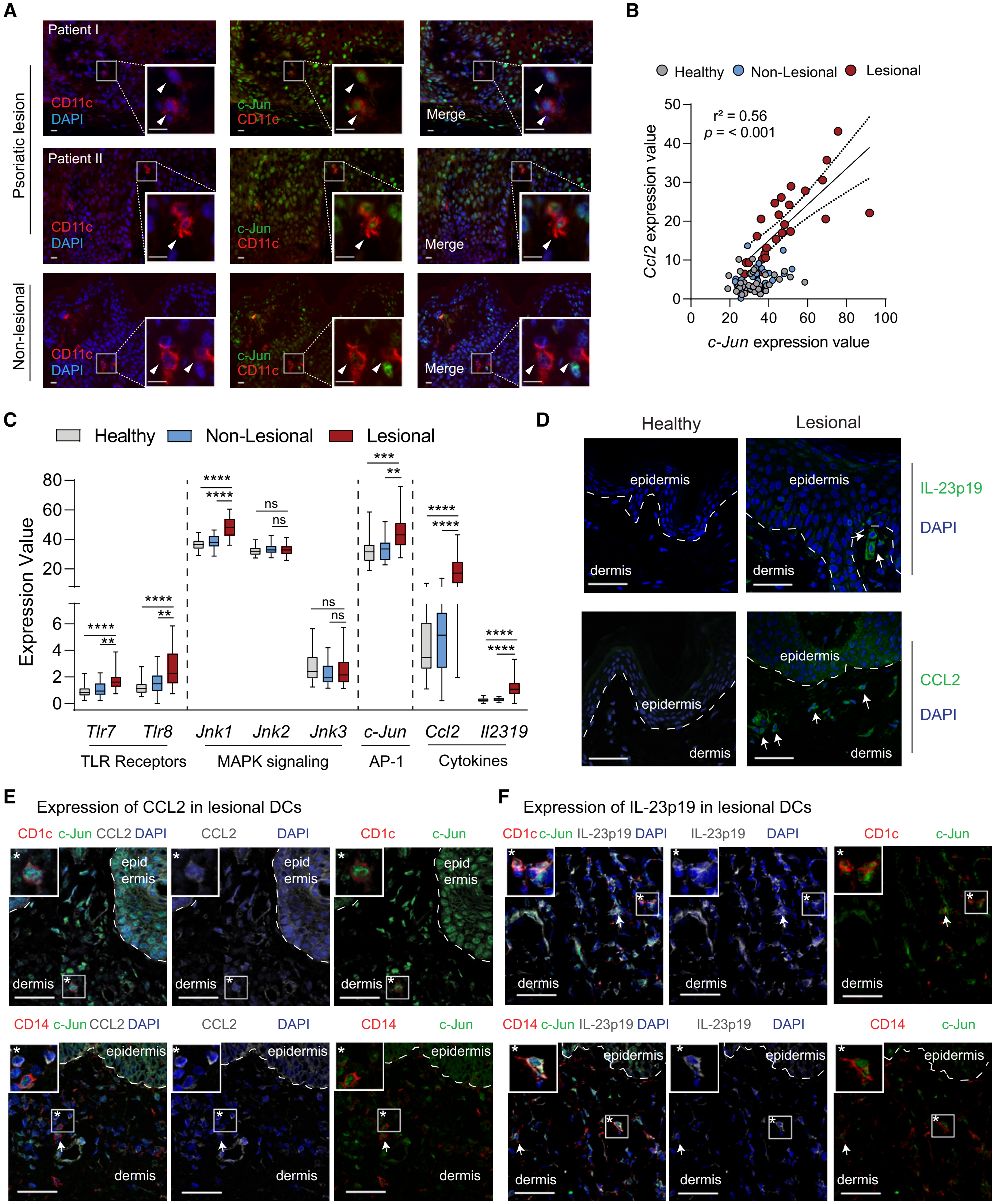

Figure EV4.

 © 2021 The Authors

Figure EV5. Role of JNK-AP-1 signaling in the maturation of human mo-DCs stimulated with R848 or LL-37-RNA-40.

A–D Human mo-DCs were pretreated with DMSO (1:1,000) or JNK Inhibitor (SP600125, 25 μM) (A, B) or AP-1 Inhibitor (T-5224, 20 μM) (C, D) for 1 h and stimulated with R848 for 24 h. Gated CD1a$^+$ cells were analyzed for surface expression of CD80 and CD86 by flow cytometry. Shown are representative histograms (A, C) and mean fluorescence intensity (MFI) (B, D) ($n = 4$ (B); representative result from 2 independent experiments is shown, $n = 7$ (D); 2 independent experiments).

E–G Human mo-DCs were stimulated with LL-37-RNA-40 complex or LL-37 or RNA-40 for 24 h. Representative flow cytometry plots and histograms are shown in (E) and mean fluorescence intensity (MFI) of CD80 and C86 is shown in (F). Expression of CCL2 and IL-23 was analyzed by ELISA (G) ($n = 7$, 2 independent experiments).

Data information: Data are shown as mean ± SEM. *P*-values were calculated by paired, two-tailed *t*-test (B, D) or one-way ANOVA with Tukey multiple comparison test (F, G). Statistical significance: ns > 0.05, *$P < 0.05$, **$P < 0.01$, ***$P < 0.001$. See Appendix Table S3 for exact *P*-values.

Source data are available online for this figure.

© 2021 The Authors

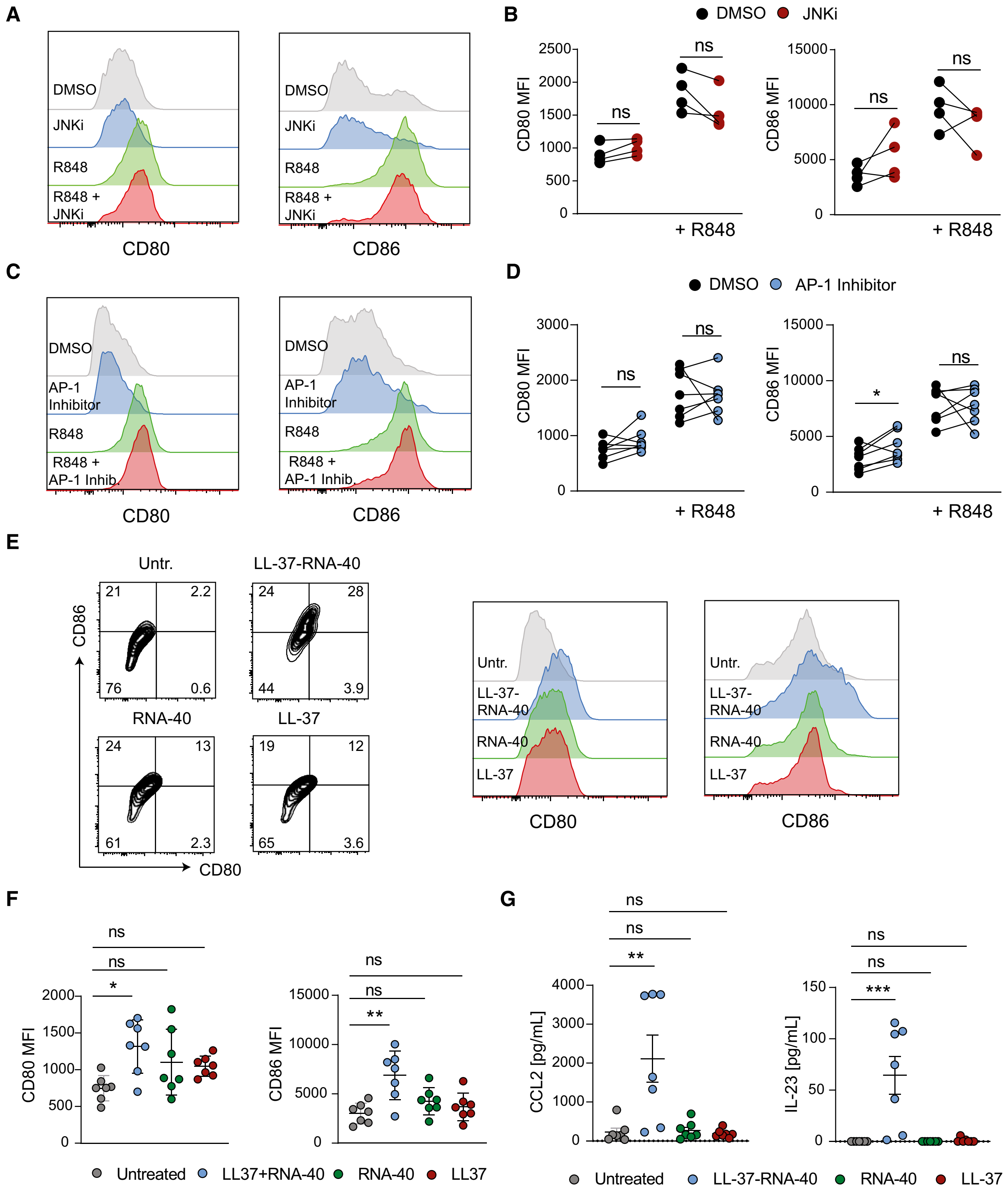

Figure EV5.

© 2021 The Authors
