# Supplementary material for: Psoriatic skin inflammation is promoted by c‐Jun/AP‐1‐dependent CCL2 and IL‐23 expression in dendritic cells
Source: EMBO Mol Med. 2021 Mar 16;13(4):e12409. doi: 10.15252/emmm.202012409 (PMC8033525; doi:10.15252/emmm.202012409)
Supplement: Supplementary file 3 — Source Data for Expanded View and Appendix [file EMMM-13-e12409-s011.zip › EV_Figures_+_Appendix/EMM-2020-12409-V5_Source_Data_EV_Fig4/EMM-2020-12409-V5_Source_Images_EVFig4E.pdf]

EV Figure 4E- Unedited Images

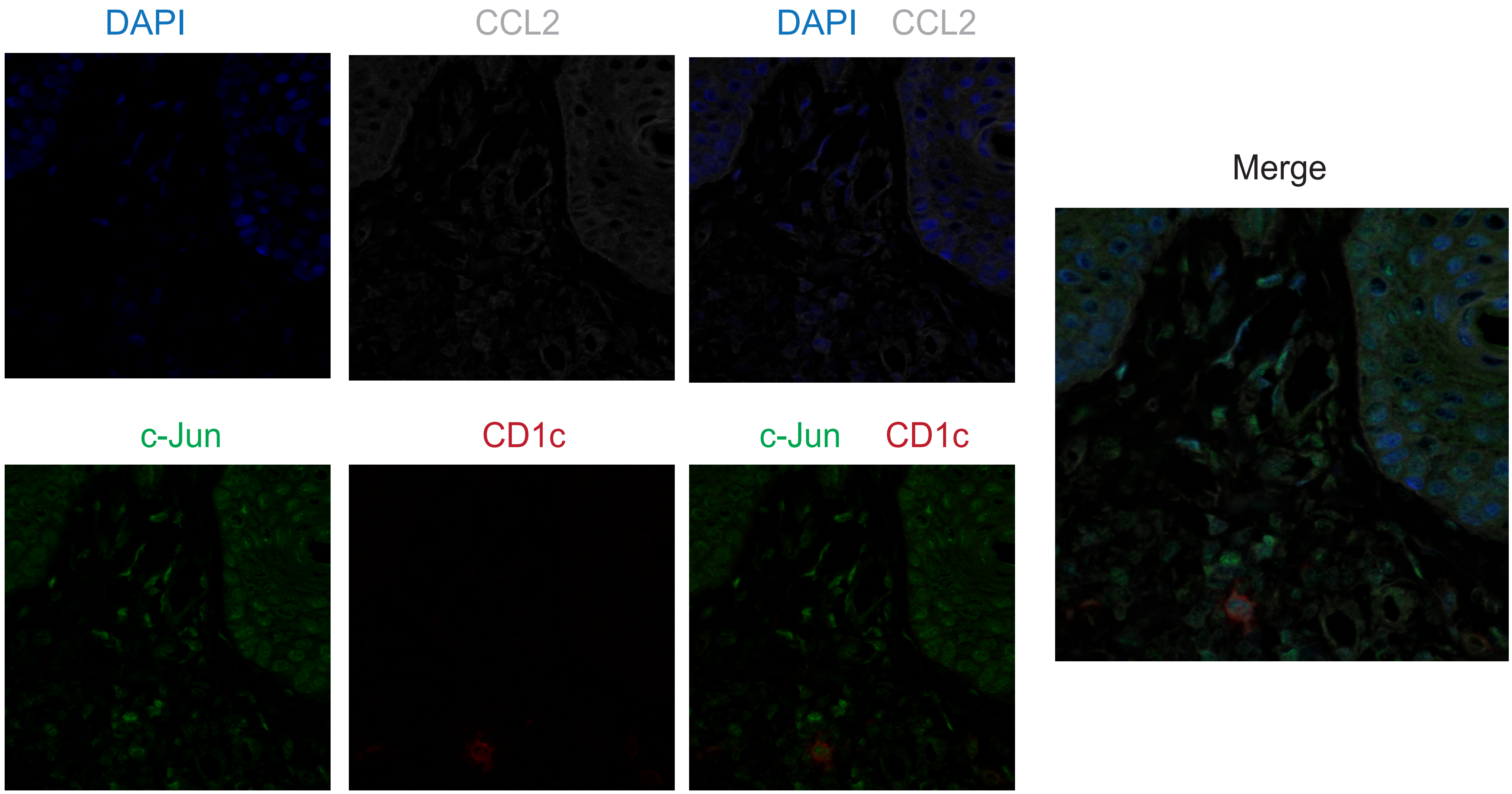

Expression of CCL2 in lesional CD1c^{+} DC

EV Figure 4E- Unedited Images

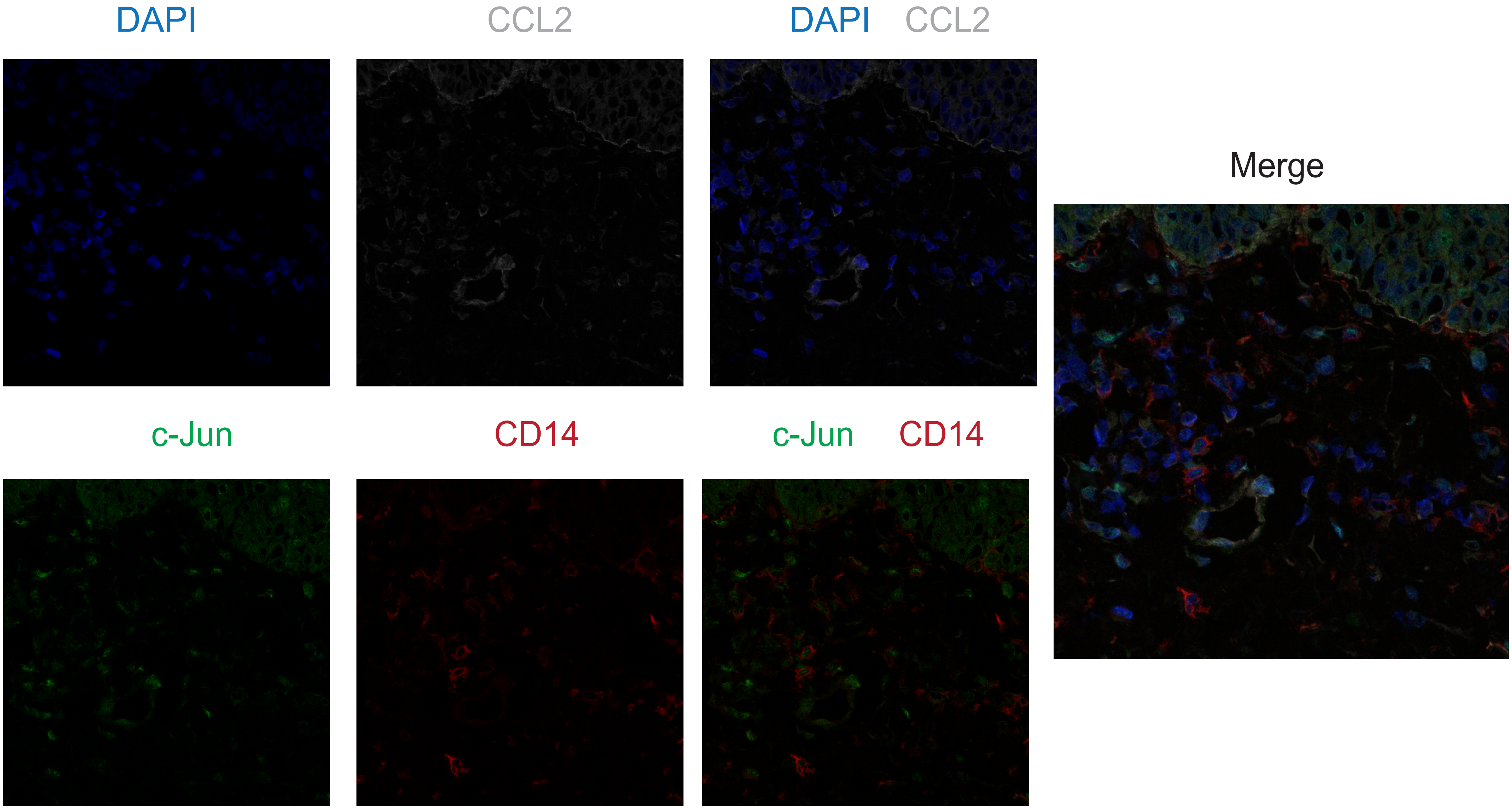

Expression of CCL2 in lesional CD14^{+} DC
