# Supplementary figures and images for: Psoriatic skin inflammation is promoted by c‐Jun/AP‐1‐dependent CCL2 and IL‐23 expression in dendritic cells
Source: EMBO Mol Med. 2021 Mar 16;13(4):e12409. doi: 10.15252/emmm.202012409 (PMC8033525; doi:10.15252/emmm.202012409)

Figure 2H - Unedited blots

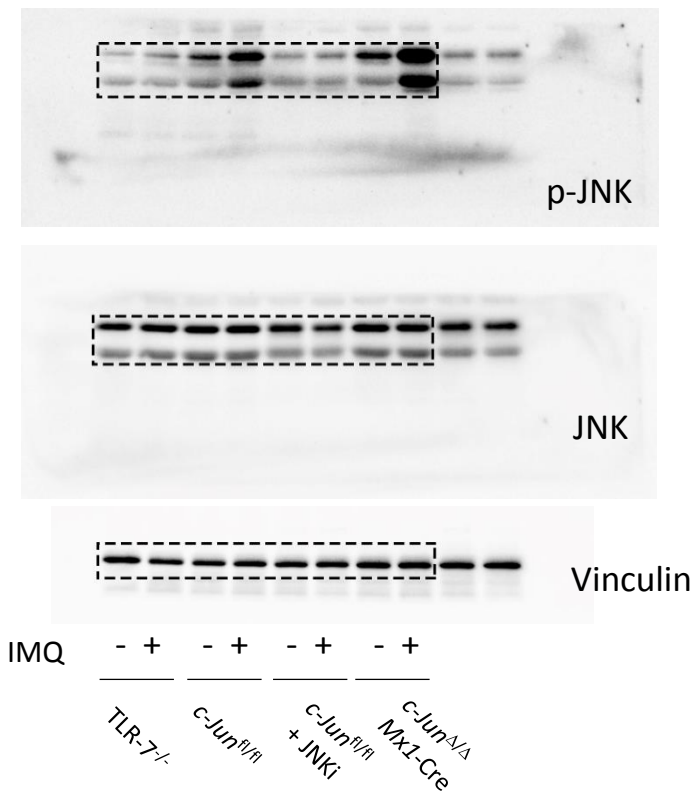

Figure 2I - Unedited blots

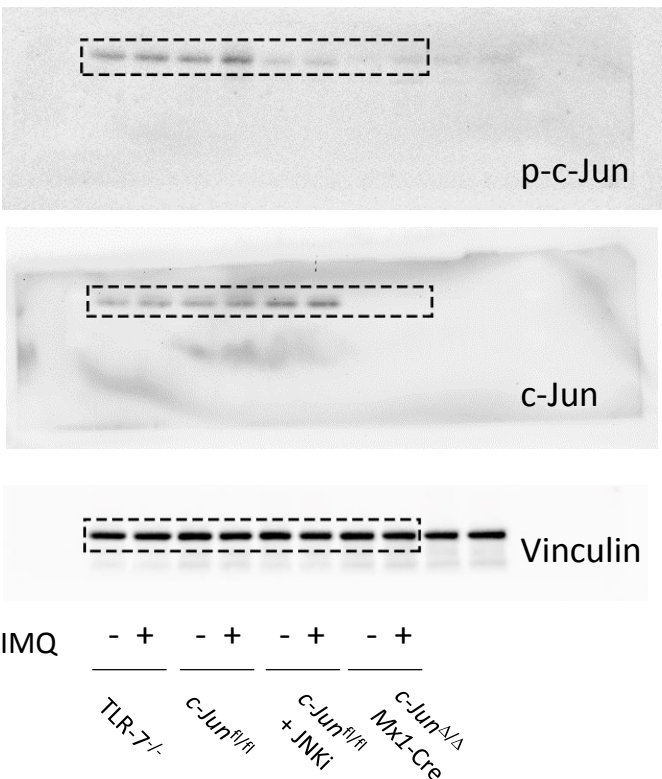

Supplement: Supplementary file 6 — Source Data for Figure 2 [file EMMM-13-e12409-s009.zip › EMM-2020-12409-V5_Source-Data_Fig2/EMM-2020-12409-V5_Unedited-Blots_Fig2.pdf]

Figure 6D- Unedited Images

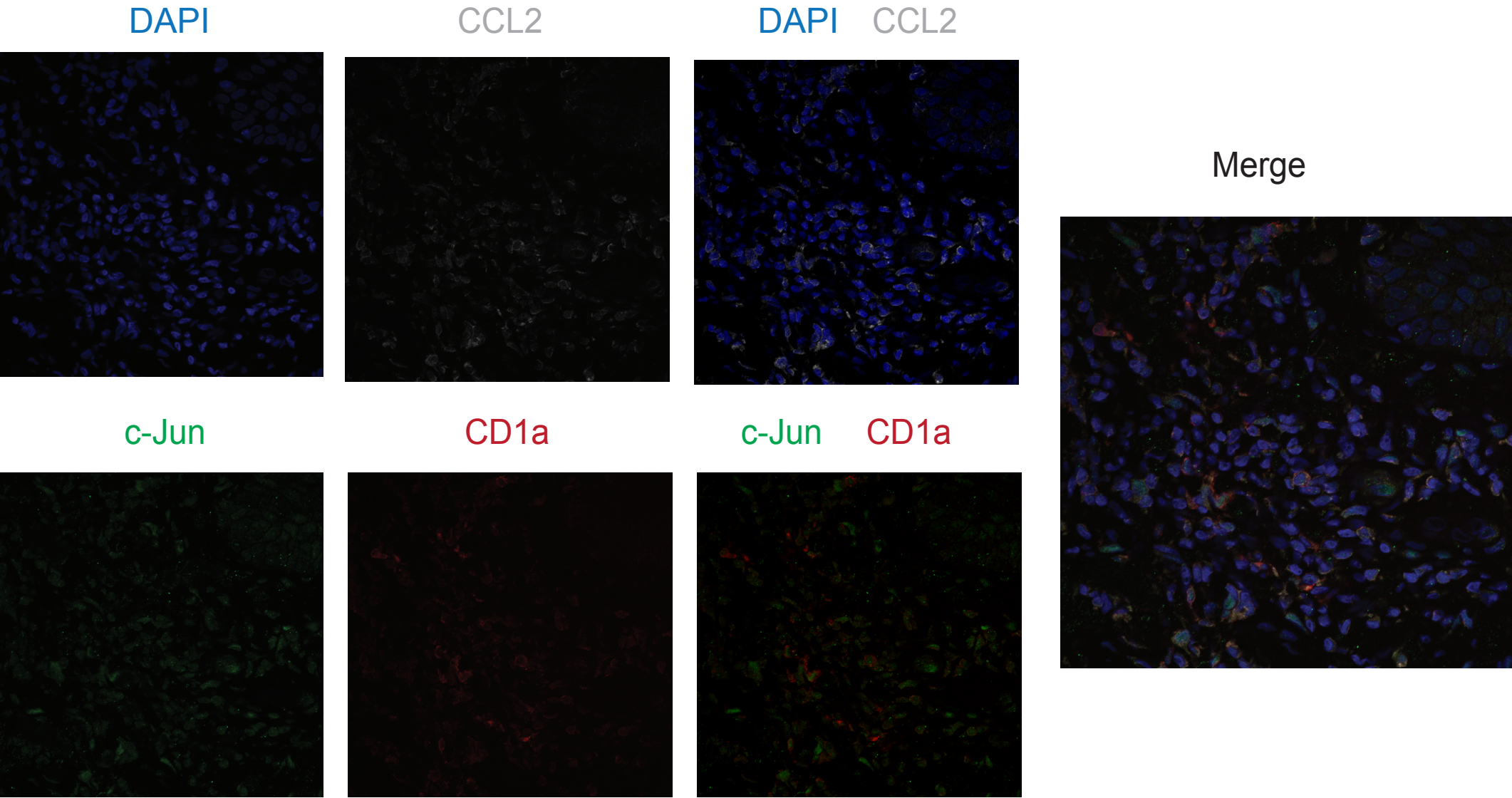

Expression of CCL2 in lesional CD1a<sup>+</sup> DC

Supplement: Supplementary file 10 — Source Data for Figure 6 [file EMMM-13-e12409-s002.zip › EMM-2020-12409-V5_Source-Data_Fig6/EMM-2020-12409-V5_Source_Images_Fig6D.pdf]

Figure 6E- Unedited Images

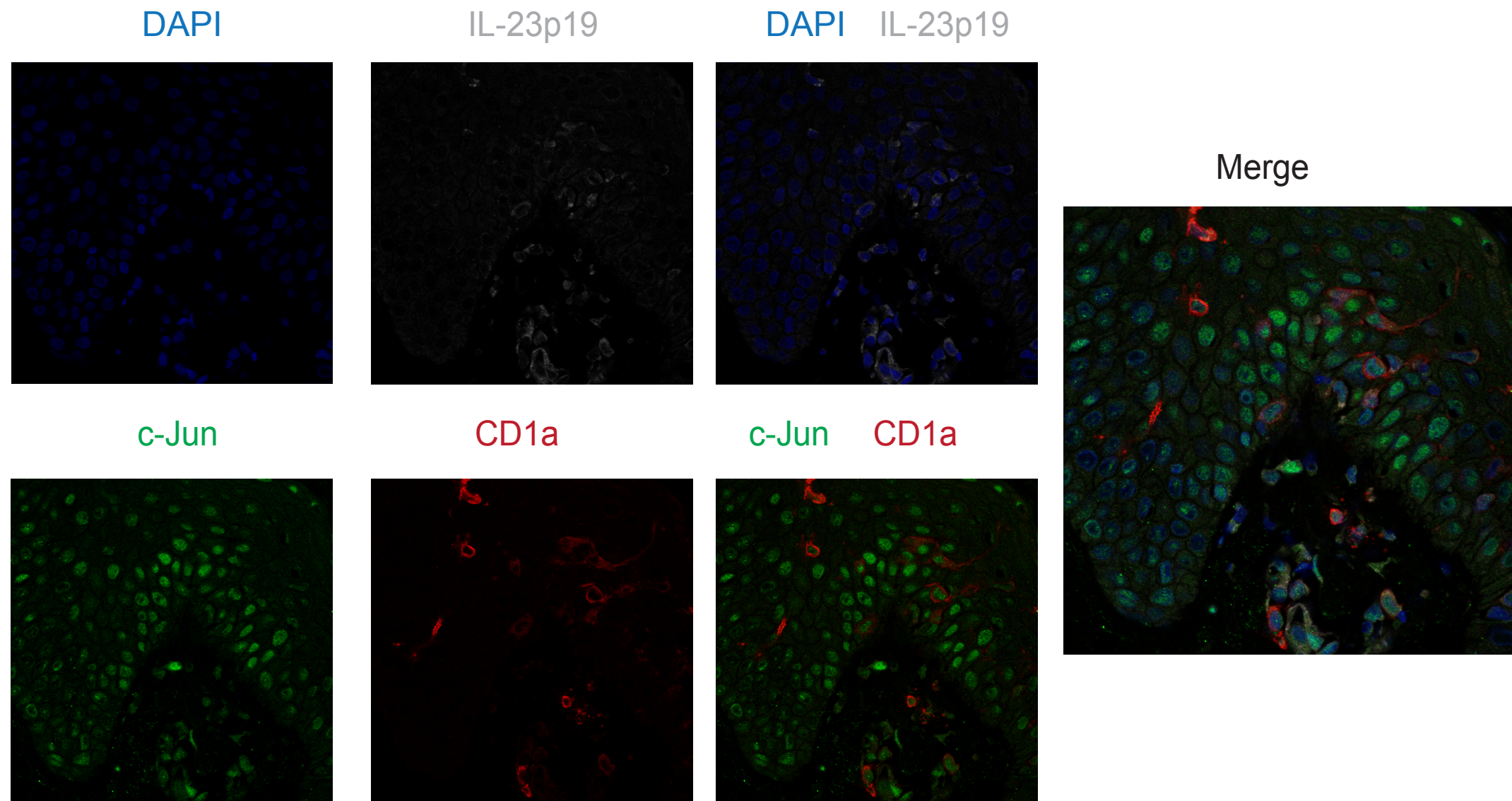

Expression of IL-23p19 in lesional CD1a<sup>+</sup> DC

Supplement: Supplementary file 10 — Source Data for Figure 6 [file EMMM-13-e12409-s002.zip › EMM-2020-12409-V5_Source-Data_Fig6/EMM-2020-12409-V5_Source_Images_Fig6E.pdf]

Figure 7A – Unedited Blots

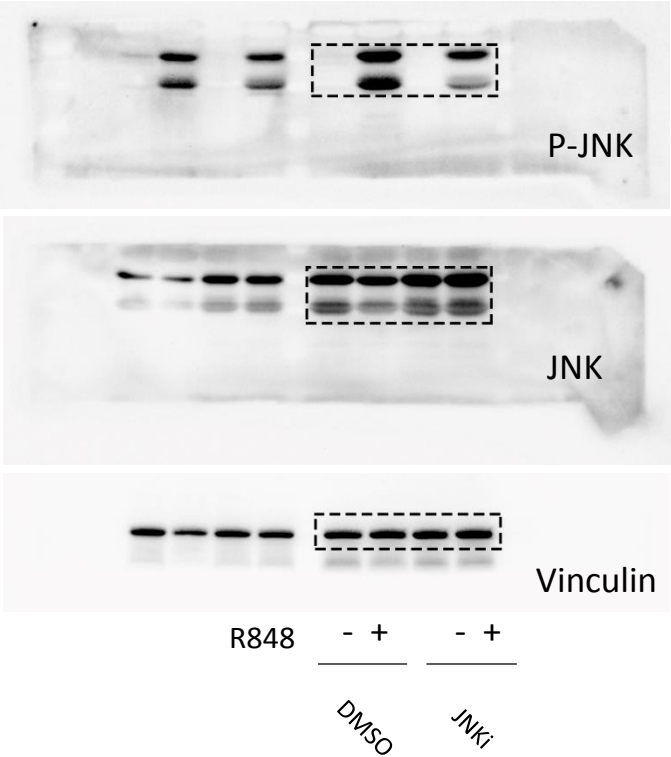

Figure 7A – Unedited Blots

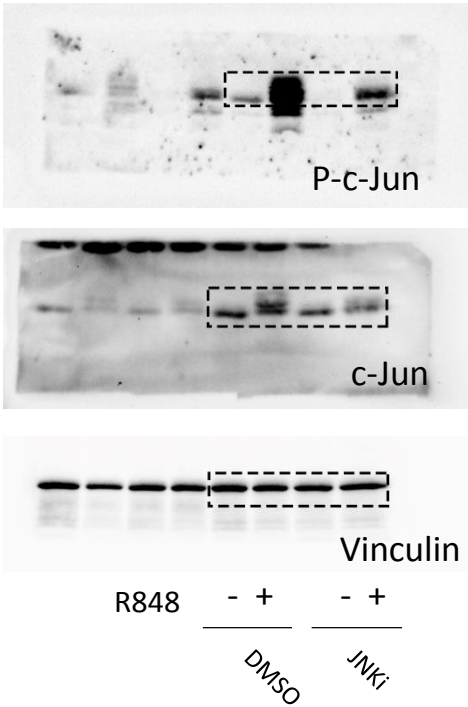

Supplement: Supplementary file 11 — Source Data for Figure 7 [file EMMM-13-e12409-s007.zip › EMM-2020-12409-V5_Source-Data_Fig7/EMM-2020-12409-V5_Unedited-Blots_Fig7.pdf]
